# Gene editing with CRISPR-Cas12a guides possessing ribose-modified pseudoknot handles

**DOI:** 10.1038/s41467-021-26989-z

**Published:** 2021-11-15

**Authors:** Eman A. Ageely, Ramadevi Chilamkurthy, Sunit Jana, Leonora Abdullahu, Daniel O’Reilly, Philip J. Jensik, Masad J. Damha, Keith T. Gagnon

**Affiliations:** 1 Department of Chemistry and Biochemistry, Southern Illinois University, Carbondale, IL USA; 2 Department of Biochemistry and Molecular Biology, School of Medicine, Southern Illinois University, Carbondale, IL USA; 3 Department of Chemistry, McGill University, Montreal, Canada; 4 Department of Physiology, School of Medicine, Southern Illinois University, Carbondale, IL USA; 5 Present Address: RNA Therapeutics Institute, University of Massachusetts Medical School, Worcester, MA USA

**Keywords:** CRISPR-Cas9 genome editing, Chemical modification, Nucleic acids

## Abstract

CRISPR-Cas12a is a leading technology for development of model organisms, therapeutics, and diagnostics. These applications could benefit from chemical modifications that stabilize or tune enzyme properties. Here we chemically modify ribonucleotides of the *As*Cas12a CRISPR RNA 5′ handle, a pseudoknot structure that mediates binding to Cas12a. Gene editing in human cells required retention of several native RNA residues corresponding to predicted 2′-hydroxyl contacts. Replacing these RNA residues with a variety of ribose-modified nucleotides revealed 2′-hydroxyl sensitivity. Modified 5′ pseudoknots with as little as six out of nineteen RNA residues, with phosphorothioate linkages at remaining RNA positions, yielded heavily modified pseudoknots with robust cell-based editing. High *trans* activity was usually preserved with *cis* activity. We show that the 5′ pseudoknot can tolerate near complete modification when design is guided by structural and chemical compatibility. Rules for modification of the 5′ pseudoknot should accelerate therapeutic development and be valuable for CRISPR-Cas12a diagnostics.

## Abstract (web summary)

Development of Cas12a for human therapeutics and diagnostics may significantly benefit from, or even require, chemical modification of its guide RNA. Here the authors show that the noncanonical 5′ pseudoknot structure of the AsCas12a crRNA guide can be heavily modified and still retain very high editing activity in cells and trans cleavage activity in vitro.

## Introduction

Clustered regularly interspaced short palindromic repeats (CRISPR) and their CRISPR-associated (Cas) enzymes represent an ancient bacterial immune system evolved to fight invading viral pathogens^1–8^. The core enzymatic component is a ribonucleoprotein (RNP) comprised of one or more Cas proteins, with at least one being a nuclease, which are associated with one or two CRISPR RNAs (crRNAs)^7–18^. After recognition of a short protospacer adjacent motif (PAM) sequence by the Cas endonuclease, base-pairing of crRNA nucleotides to target DNA then guides sequence-specific and site-specific endonucleolytic cleavage of DNA phosphodiester bonds^10,19–21^.

Cas9 from *Streptococcus pyogenes* (*Sp*Cas9) and Cas12a, also known as Cpf1, from *Acidominococcus* species (*As*Cas12a) are among the most heavily co-opted and engineered CRISPR-Cas systems for creation of model organisms and development of human therapeutics^22–33^. They both efficiently induce DNA double-strand breaks and guide genome editing in mammalian cells^16,22,23,33–38^, utilize a single Cas enzyme and can function with a single-guide RNA (sgRNA), although Cas9 functions as a dual-guide RNA in nature^8,16,23,33^. While these enzymes can ultimately result in similar gene editing outcomes, such as genetic knockout or donor DNA sequence knock-in, they are quite distinct. Cas12a is a smaller protein that naturally utilizes a single crRNA guide (39 nucleotides), less than half the size of the commonly used artificial Cas9 sgRNA (99 nucleotides)^16,20,39^. Cas12a recognizes thymine (T)-rich PAMs, whereas *Sp*Cas9 uses guanine (G)-rich PAMs^8,16,21,40^. The Cas12a crRNA adopts a pseudoknot structure at its 5′ end that creates a “handle” for Cas12a to bind, as opposed to a series of stem structures for *Sp*Cas9^20,39^.

A unique property of Cas12a is *trans* activity, which is a non-sequence-specific single-stranded DNA cleavage (ssDNase) activity with high catalytic turnover^41,42^. *Trans* activity requires that the Cas12a RNP first bind to and cut a target DNA strand, which is referred to as *cis* activity. *Trans* activity has been exploited to create molecular diagnostics that can detect very small amounts of DNA and amplify the detection signal through cleavage of ssDNA substrates that can be detected, such as by fluorescence, antibodies on test strips, and nanopores^41,43,44^. The digestion of the ssDNA reporter by *trans* activity is intrinsically tied to the *cis* cleavage of the specific target DNA because both mechanisms use the same catalytic residues^45,46^. Mutation of neighboring amino acids or REC (recognition lobe) linker and lid regions of Cas12a, however, could allow *trans* but not *cis* activity when Cas12a was bound to a complementary single-strand DNA target^46^. However, it appears that *cis* and *trans* activity cannot be fully uncoupled for recognition of double-stranded targets via *trans* activity without cis cleavage of the target strand^45,46^.

CRISPR-Cas systems hold tremendous promise for revolutionizing medicine, particularly through improved gene therapy or gene-targeted approaches^26,47–56^. To achieve this potential, however, various challenges must be overcome. These include long-term safety, delivery, and efficacy^26,37,52–54,56–59^. Lipid nanoparticles are a leading technology being developed for delivery of Cas enzymes, either as protein or as mRNA that encode the Cas protein, along with their cognate crRNA guides^54,60–64^. In principle, this may allow greater spatial and temporal control over tissue and cell targeting^63^ and provide a better window of safety when compared to therapeutic delivery approaches that involve genetically encoded expression of the CRISPR-Cas system, such as adeno-associated viruses^64^. However, RNA is not an ideal drug due to its size and lability—it is quickly degraded in the body. Chemical modification has emerged as a practical necessity to stabilize and control the half-life, bioavailability, and activity of RNA and nucleic acid drugs^65,66^. Chemical modification has been a pillar in the growing success of other nucleic acid-based therapeutics, including antisense oligonucleotides (ASOs) and small interfering RNAs (siRNAs)^58,66,67^. The lessons learned from these technologies can be applied to CRISPR-based therapeutics. The degree of chemical modification required for crRNA guides is unclear and may depend on delivery or target tissue. Nonetheless, ultimate control over stability, protein interaction, enzyme activity, and pharmacology would ideally be achieved by understanding what chemical modifications are tolerated at every position within a crRNA guide^47,61,66,68–71^.

Previous investigations of CRISPR-Cas guide RNA chemical modification have primarily focused on improving therapeutic gene editing by increasing nuclease resistance, understanding biochemical limitations, and reducing off-target editing. Minimal addition of modified nucleotides or inter-nucleotide linkages at the guide RNA 5′ and 3′ termini have been shown to maintain or increase editing, presumably through reduced nuclease sensitivity. These have included 2′-*O*-methyl with phosphorothioate (PS-2′OMe) or 2′-*O*-methyl-3′-thiophosphonoacetate (2′OMe thioPACE) for Cas9^72,73^. In the Cas12a system, 3′ end capping has been explored with 2′-F, 2′-*O*-methyl, unlocked nucleic acid (UNA), locked nucleic acid (LNA), and phosphorothioate (PS)^74^ while 5′ end capping has been explored by addition of short MS or DNA-PS modified sequences onto the 5′ handle^75^. Interestingly, extension of the 3′ end with 2′-*O*-methyl nucleotides^76^ or the 5′ handle with DNA-PS^77^ has also been reported to improve editing or increase the kinetics of *trans* cleavage activity for Cas12a, respectively.

More complete or systematic modification of Cas9 and Cas12a guide RNAs has been explored using a variety of chemistries. DNA (2′-deoxy) has been reported to alter enzyme activity or specificity while helping to probe 2′-hydroxyl requirements of the enzyme^69,78–80^. A broader array of modifications, like 2′-F, 2′-*O*-methyl, bridged nucleic acid (BNA), UNA, LNA, 2′F-arabinonucleic acid (FANA), and PS have been tested alone or in combination as well^61,70,71,81–84^. These studies largely demonstrated that much of the guide could be modified, with moderate to high editing preserved, but particular positions, such as seed regions or individual residues, required RNA nucleotides. Specificity could also be improved with careful placement of modifications in the guide region^78,79,83,84^. For Cas9, retention of editing activity has been suggested to correlate with conserved polar contacts between the protein and the hydroxyl at the ribose 2′ position and the need to retain A-form-like helical architecture^69–71,80^. For these apparently conserved positions between Cas9 and its guide, RNA cannot be readily replaced.

For Cas12a, attempts to fully modify the crRNA guide have proven unsuccessful^74,78,81^. However, the 5′ pseudoknot handle posed the greatest challenge as it appeared to tolerate very little modification to the ribose, suggesting that successful modification of this unique structure would be the major limitation to greater modification of Cas12a guides for therapeutic development. To address this bottleneck, we took a rational, structure-guided approach with respect to predicted 2′-hydoxyl (2′-OH) polar contacts.

In this work, we utilized chemical modifications that specifically explored ribose and 2′-OH properties. We successfully modified the 5′ pseudoknot and obtained very robust gene editing when as few as 6 out of 19 residues remained unmodified. The key positions that remained resistant to modification correlated with predicted 2′-OH polar contacts within the Cas12a-crRNA crystal structure^20,39^. The bridging phosphodiester bonds 3′ to these residue positions could further be modified to PS and retain very high *cis* and *trans* cleavage activity, as well as gene editing. When crRNAs with chemically modified 5′ pseudoknot handles were screened for *trans* cleavage activity, some induced differential activities like high *trans* activity with little or no *cis* dsDNA target cleavage. These results were relegated to the high catalytic turnover of *trans* activity even when very modest *cis* cleavage occurred. Together, these results advance efforts toward chemical modification of *As*Cas12a guides for therapeutic editing and potential diagnostic applications.

## Results

### Probing 2′-OH contacts and A-form preference with 2′-deoxyribose

**Fig. 1:**
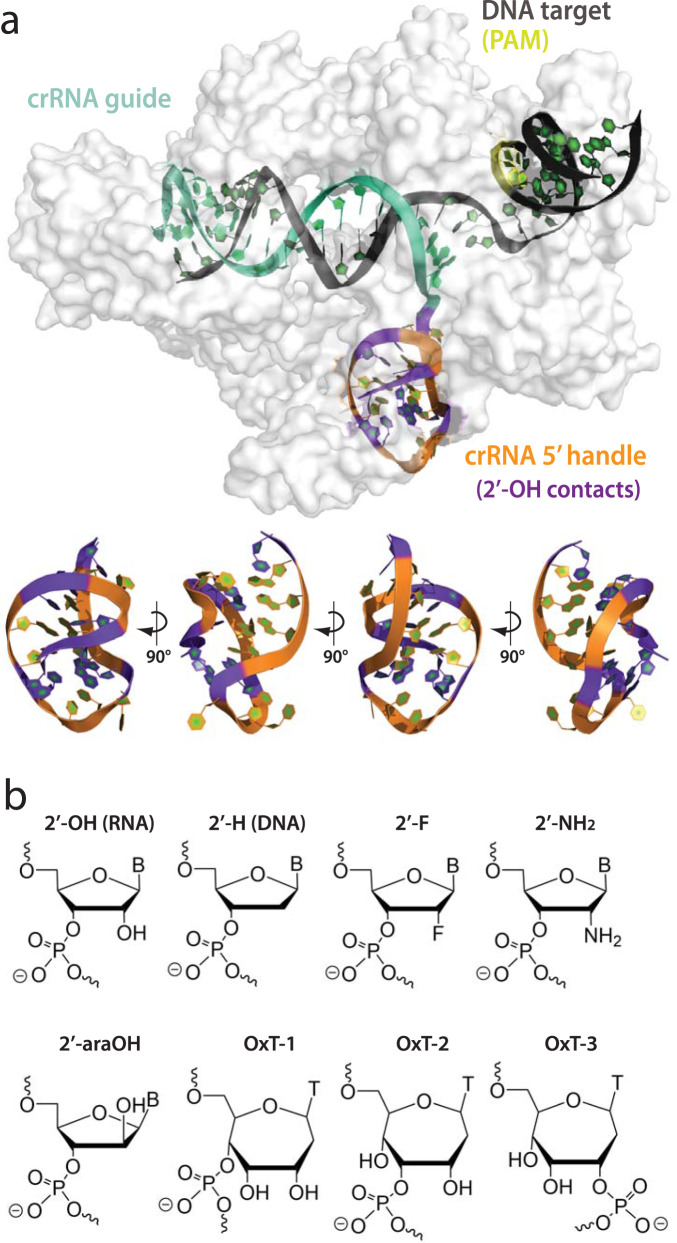
Structural overview of AsCas12a ribonucleoprotein complex, the crRNA 5′ pseudoknot, and chemical modifications incorporated into the pseudoknot. **a** *As*Cas12a 5′ handle pseudoknot structure (PDB ID 5B43). Cas12a protein is rendered as a surface (light gray), DNA target as dark gray, PAM sequence in yellow, crRNA guide in teal, 5′ handle in orange, and 5′ handle nucleotides that make 2′-OH polar contacts within the structure in purple. **b** Chemically modified ribose analogs and substitutes used in this study.

*Acidominococcus* species Cas12a (*As*Cas12a) and a 39-nucleotide crRNA were used for this study. The crRNA can be structurally and functionally divided into two domains: a 19-nucleotide 5′ handle with a pseudoknot structure that anchors crRNA binding to Cas12a and a 20-nucleotide 3′ guide region that base-pairs to complementary target strand DNA (Fig. 1a). A crystal structure of *As*Cas12a has enabled prediction of potentially important polar contacts or hydrogen bonds between *As*Cas12a and the RNA via 2′-hydroxyl (2′-OH) groups in the pseudoknot structure, as well as intramolecular 2′-OH RNA-RNA contacts^20^. The importance of RNA A-form helical structure and 2′-OH chemistry has been partially investigated for pseudoknot stability and protein interaction^85,86^, but not in the context of CRISPR-Cas systems. The unique noncanonical nature of the Cas12a 5′ pseudoknot, as well as previous attempts at modification^74,78,81^, suggested that it would be challenging to modify for therapeutic development. To focus on the pseudoknot structure and uncouple it from guide region effects, we maintained an entirely native RNA ribose (2′-OH) guide region throughout most of this study. This was accomplished through splint ligation of a 5′ phosphorylated guide RNA and chemically modified pseudoknot RNAs using T4 DNA ligase^87^ (Supplementary Fig. 1) or direct solid-phase chemical synthesis of the entire crRNA when modification schemes prevented efficient enzymatic ligation. Enzymatic ligation facilitated more cost-effective and modular production of full-length crRNAs. To investigate the role of the ribose 2′-OH and A-form structural preferences in 5′ pseudoknot structure-function for *As*Cas12a, we substituted RNA nucleotides with 2′-deoxy (DNA), 2′-fluoro (2′-F), oxepane (OxN), 2′-arabino (2′-araOH), and 2′-amino (2′-NH_2_) residues (Fig. 1b).

**Fig. 2:**
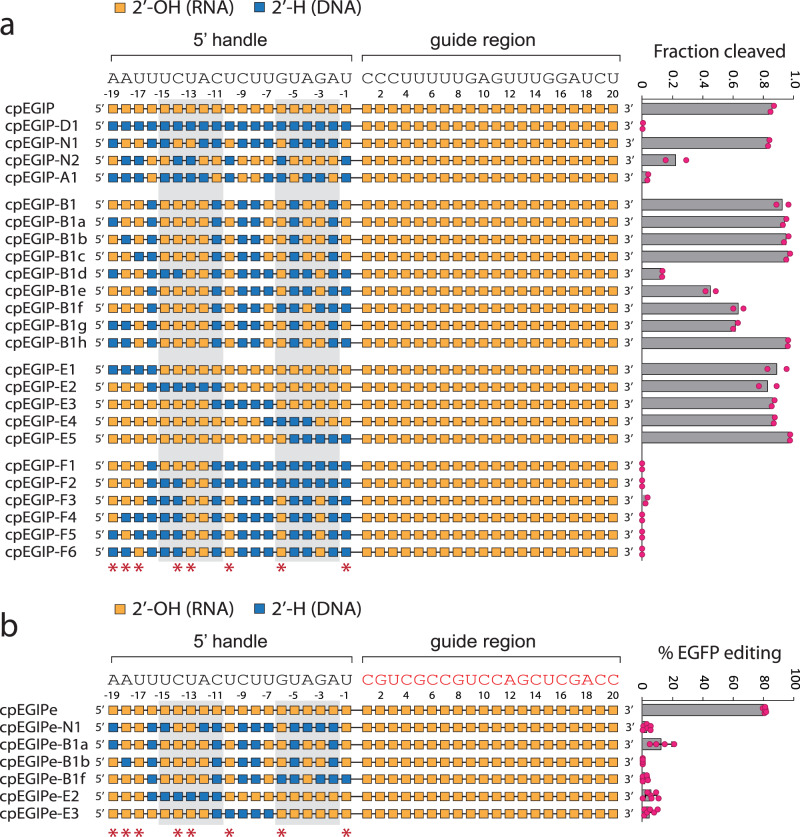
In vitro cleavage and cell-based editing activity of *As*Cas12a using crRNA guides modified with 2′-deoxyribose (DNA) nucleotides. DNA modification patterns are illustrated, with corresponding mean in vitro cleavage activity (*n* = 2) (**a**) or mean cell-based editing activity (*n* = 6) (**b**) shown to the right. Error bars are standard deviation and replicates are biological. For both panels, nucleotides of the 5′ handle that are predicted from structural data to make polar contacts through 2′-OH hydrogen bonding are indicated with red asterisks below. The base-paired stem nucleotides of the 5′ handle pseudoknot are enclosed with light gray boxes. In vitro cleavage and cell-based editing assays utilize the distinct guide sequences that are shown above each schematic. Source data are provided as a Source Data file.

While DNA nucleotides can probe the importance of 2′-OH contacts, they introduce conformational flexibility and can therefore also probe the importance of A-form structural preferences^69,88,89^. Pseudoknots are noncanonical RNA structures that have also been generated with single-stranded DNA^90,91^. Thus, we initially synthesized a crRNA with all nucleotides in the pseudoknot substituted with DNA (cpEGIP-D1). No sequence-specific (*cis*) cleavage activity was observed for this design in vitro, indicating that either 2′-OH contacts or A-form helical structure, or both were necessary for Cas12a RNP assembly or enzyme activity (Fig. 2a). Based on an *As*Cas12a crystal structure^20^, we identified putative critical 2′-OH contacts in the pseudoknot structure at positions -1, -6, −10, -13, -14, -17, -18, and -19. Converting these positions back to RNA, except -19, (cpEGIP-N1) completely rescued in vitro cleavage activity. These results, along with inspection of the Cas12a crystal structure, suggested to us that the 2′-OH at residue -19 may not be a critical polar contact that impacts activity. Conversely, an all-RNA pseudoknot with the same seven potentially critical 2′-OH contact positions (omitting position -19) replaced with DNA (cpEGIP-N2) only provided a quarter of the normal *As*Cas12a activity. Thus, these results supported the critical role of 2′-OH contacts for either pseudoknot structure or protein interaction. However, the observation that cpEGIP-N2 still conferred cleavage activity with no 2′-OH groups at the predicted critical positions also suggested that A-form helical structure or C3′-*endo* sugar pucker may play an important, albeit potentially lesser role. Indeed, adding additional DNA nucleotides to cpEGIP-N2 to create cpEGIP-A1, which possessed only six RNA residues, abrogated cleavage activity. The activity of cpEGIP-N2, although low, also suggested that pseudoknot 2′-OH contacts may not be obligatory for activity.

To further test the role of individual positions and explore RNA–DNA combinations, we synthesized and tested several more crRNAs. Starting with a design that preserved the putative critical 2′-OH contacts and included more RNA residues (cpEGIP-B1), we replaced -19, -18, and -17 positions individually (cpEGIP-B1a, -B1b, and -B1c). None of these substitutions were detrimental to activity (Fig. 2a). We then added more DNA residues, including at positions -14 and -1. This resulted in substantially reduced activity (cpEGIP-B1d). Adding RNA back to positions -14 and -15 improved activity (cpEGIP-B1e). Placing a DNA nucleotide at the putative critical 2′-OH position -6, as well as -3, showed even greater enzyme activity (cpEGIP-B1f), suggesting that -6 is more tolerant of 2′-OH loss. Comparing two pseudoknots with the same DNA substitution pattern but with or without DNA at position -1 (cpEGIP-B1g and -B1h) revealed that DNA at -1 was not only well tolerated but also seemed to enhance the activity. These results suggested that no specific critical 2′-OH position was necessarily more important than another and instead that their cumulative loss is additive and negatively impacts enzyme activity.

To test both specific positions and cumulative DNA effects, we systematically walked overlapping blocks of 4–6 DNA nucleotides across the pseudoknot sequence (cpEGIP-E1 through -E5). *As*Cas12a exhibited high activity with all these crRNAs (Fig. 2a). This result supports the notion that no individual RNA position is essential and high activity can be achieved in vitro with DNA substitutions if sufficient RNA nucleotide content is preserved. Indeed, a new series of crRNAs where we added increasing numbers of DNA residues, including at potentially critical 2′-OH contact positions (cpEGIP-F1 through -F6), abrogated *As*Cas12a activity. These results support the important role of A-form helical preference or reduced flexibility introduced by C3′-*endo* sugar pucker.

Previous studies by us and others have determined that specific native 2′-OH ribose chemistry (RNA nucleotides) is not necessary for in vitro cleavage but is required for efficient gene editing in cells by CRISPR-Cas9 from *S.* *pyogenes*^70,71,80^. Critical 2′-OH contact positions lie in the *Sp*Cas9 crRNA guide (spacer) seed region and the repeat region proximal to the guide^70,71^. Thus, they are localized in the center of the *Sp*Cas9 crRNA. Furthermore, RNA–DNA chimeric Cas9 crRNAs composed of DNA with only RNA at the critical 2′-OH positions were highly active in vitro^69^. To determine if a similar phenomenon existed for the 5′ pseudoknot of Cas12a, we selected several RNA–DNA chimeric pseudoknot crRNAs with high activity in vitro and tested their ability to knock out, and therefore edit, an EGFP gene. We started with HEK293T cells stably expressing EGFP^79^ and transduced them with a 3xNLS-*As*Cas12a-expressing lentivector followed by selection (Supplementary Fig. 2). crRNAs were lipid-transfected into these custom HEK293T cells constitutively expressing EGFP and *As*Cas12a. For cell-based editing experiments, 5′ pseudoknots were ligated to a different guide sequence (hence the designation cpEGIPe) designed to target the integrated EGFP gene (Supplementary Fig. 2). Our native all-RNA control routinely generated about 70–80% knockout of EGFP when measured by flow cytometry 5 days after transfection (Fig. 2b and Supplementary Fig. 2d). However, the RNA–DNA chimeric pseudoknots generated little or no editing activity. Notably, cpEGIPe-N1 was inactive despite preserving RNA at the putative critical 2′-OH positions. Only cpEGIPe-B1a provided modest editing (approximately 10%), perhaps due to better conservation of A-form structure by additional RNA nucleotides. Thus, as had been observed with *Sp*Cas9 crRNAs, retaining 2′-OH at potentially critical positions appeared to be necessary but not sufficient to provide gene editing activity^70,71^. These results highlight the more complex nature of editing in cells and support the complementary role of both A-form helical structure and 2′-OH contacts within the Cas12a crRNA 5′ pseudoknot.

### 2′-OH substitutions and RNA mimics in the 5′ pseudoknot

**Fig. 3:**
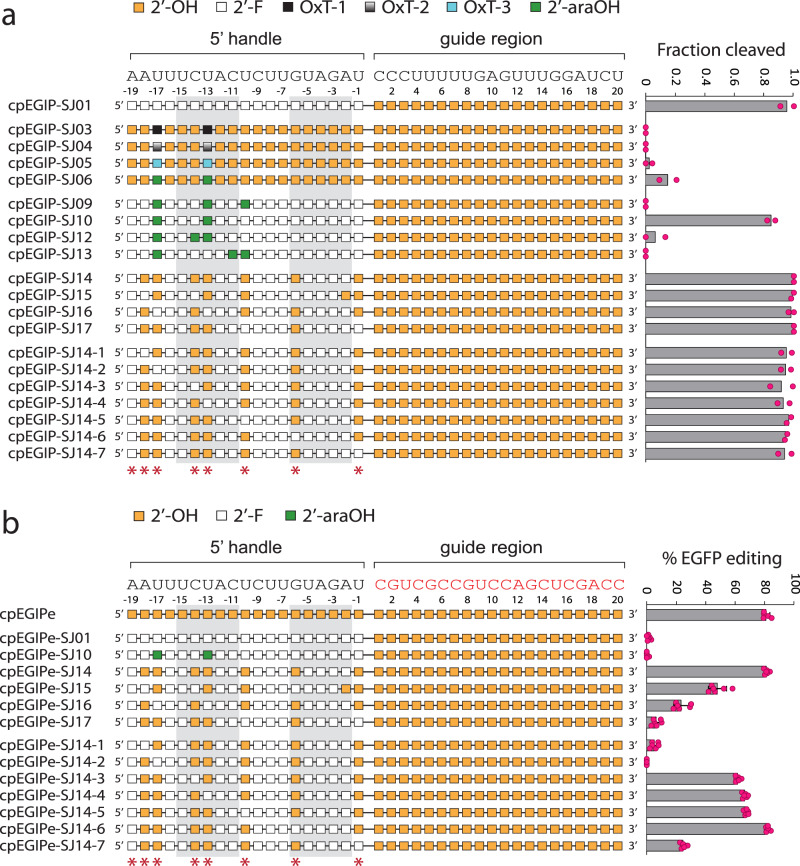
In vitro cleavage and cell-based editing activity of *As*Cas12a using crRNA guides modified with 2′-F, oxepane nucleotides, and 2′-araOH. Chemical modification patterns are illustrated with corresponding mean in vitro cleavage activity (*n* = 2) (**a**) or mean cell-based editing activity (*n* = 6) (**b**) shown to the right. Error bars are standard deviation and replicates are biological. For both panels, nucleotides of the 5′ handle that are predicted from structural data to make polar contacts through 2′-OH hydrogen bonding are indicated with red asterisks below. The base-paired stem nucleotides of the 5′ handle pseudoknot are enclosed with light gray boxes. In vitro cleavage and cell-based editing assays utilize the distinct guide sequences that are shown above each schematic. Source data are provided as a Source Data file.

To further investigate the nature of A-form helical preference and ribose chemical compatibility in 5′ pseudoknot structure–activity, we substituted RNA nucleotides with additional modified ribose nucleotides or sugar replacements. The first design was a complete substitution of the pseudoknot with 2′-fluoro-ribose (2′-F) (cpEGIP-SJ01). This fully modified pseudoknot provided very high cleavage activity, similar to or higher than the native all-RNA control (Fig. 3a). The robust activity of this modified pseudoknot indicated that the hydroxyl chemistry at the 2′ position was completely dispensable for intrinsic enzyme activity. Importantly, 2′-F is known to stabilize C3′-*endo* sugar pucker and strongly favor A-form helical structure^92,93^. However, when tested for cell-based editing activity cpEGIP-SJ01 was completely inactive (Fig. 3b). 2′-F nucleotides are potentially able to accept hydrogen bonds but not donate them, making them an incomplete replacement for RNA where 2′-OH contacts may be critical. While the combination of stable structure, mimicry of RNA properties, and maintenance of sufficient 2′ contacts^93^ have enabled 2′-F to substitute for 2′-OH in vitro, cell-based editing is apparently more sensitive and likely requires retention of specific 2′-OH contacts.

To specifically investigate positions with predicted 2′-OH contacts, we synthesized crRNAs with RNA or 2′-F pseudoknots containing oxepane nucleic acid (OxN) and 2′-araOH in putative critical 2′-OH positions. OxN is a seven-membered ring structure that can be synthesized with multiple hydroxyl groups and a phosphodiester linkage at different ring positions^94^. We synthesized thymidine (OxT) nucleotide replacements with the phosphodiester linkage at three different positions, designated OxT-1, -2, and -3. These were then incorporated into the pseudoknot sequence at putative critical 2′-OH positions -13 and -17 during solid-phase synthesis. The incorporation of OxN (cpEGIP-SJ03, -SJ04, and -SJ05) completely abrogated *As*Cas12a activity, indicating that certain OxN properties, such as the bulkiness of the seven-membered ring or conformational restraints, may simply be incompatible despite providing hydroxyl groups at various positions (Fig. 3a). Based on these results we chose to not proceed further with OxN.

Arabinose nucleotides (2′-araOH) are stereoisomers of RNA that place the hydroxyl group at the 2′ position on the opposite face of the ribose ring. While arabinonucleosides retain a 2′-OH group, the “up” orientation steers the arabinose sugar to adopt a C2′-*endo* (DNA-like) conformation^95^. When placing 2′-araOH at -13 and -17 positions (cpEGIP-SJ06), some enzyme activity was retained. We therefore chose to explore more 2′-araOH substitutions but in the background of a 2′-F pseudoknot, reasoning that the strong A-form preference of 2′-F might offset structural preferences of 2′-araOH. Incorporating more than two 2′-araOH (cpEGIP-SJ09, -SJ12, and -SJ13) severely reduced or eliminated enzyme activity (Fig. 3a). Only cpEGIP-SJ10, with 2′-araOH at positions -13 and -17, supported high in vitro *As*Cas12a cleavage activity. These results suggest that 2′-F may be successfully combined with other modifications that would otherwise be too detrimental to support high activity. Despite the promising in vitro activity of cpEGIP-SJ10, it provided no editing in cells (Fig. 3b).

### 2′-Fluoro, 2′-OH, and 2′-amino combinations in the 5′ pseudoknot

The high in vitro activity of 2′-F and its ability to counteract detrimental effects of 2′-araOH incorporation prompted us to create pseudoknots with a combination of 2′-F and RNA nucleotides that might improve cell-based editing. We made an all-2′-F pseudoknot with RNA nucleotides at putative critical 2′-OH positions (except -19). This design, cpEGIP-SJ14, supported very high in vitro cleavage (*cis*) activity (Fig. 3a). Importantly, when we transfected this same design (cpEGIPe-SJ14) into HEK293T cells stably expressing EGFP and *As*Cas12a, we observed robust editing of EGFP at levels as high or greater than our native RNA control cpEGIPe (Fig. 3b). Other designs that further reduced the number of RNA nucleotides to 5 and incorporated 2′-F at putative critical positions (cpEGIP-SJ15, -16, and -17) did not affect in vitro cleavage. However, the analogous designs targeting cellular EGFP showed varying degrees of reduced editing efficiency (Fig. 3b). This result supports the critical role of the putative 2′-OH positions and the value of modifications that retain strong A-form-like helical structure for high editing efficiency.

Different degrees of editing among RNA and 2′-F combinations suggested that some 2′-OH interactions were more critical than others, but clear patterns were difficult to discern. Therefore, we created a new series of cpEGIP-SJ14 designs where each of the seven remaining RNA nucleotide were individually substituted with a 2′-F residue. While all designs were highly active in vitro (Fig. 3a), clearer trends were observed for cell-based editing. Placing 2′-F at -18 and -17 in the SJ14 design (cpEGIPe-SJ14-1 and -SJ14-2) was very detrimental to editing, reducing it to below 10% (Fig. 3b). Positions -14, -13, and -10 could be individually replaced by 2′-F (cpEGIPe-SJ14-3, -SJ14-4, and -SJ14-5) with only mild reductions in editing compared to the RNA control. Surprisingly, replacement at position -6 (cpEGIPe-SJ14-6) resulted in very robust editing of ~90%, suggesting that this position is less sensitive and can be replaced readily with 2′-F. This result reinforced our initial finding with DNA replacements that suggested position -6 does not depend heavily on 2-OH contacts or chemistry (Fig. 2a). Finally, replacing position -1 with 2′-F created strong reductions in gene editing activity. Walking 2′-F through the remaining putative 2′-OH positions identified a crRNA with a pseudoknot having only six RNA residues out of 19 (cpEGIPe-SJ14-6) that produced *As*Cas12a editing activity as high or greater than a native crRNA. It also identified higher sensitivity to 2′-F substitution at the terminal ends of the pseudoknot sequence.

**Fig. 4:**
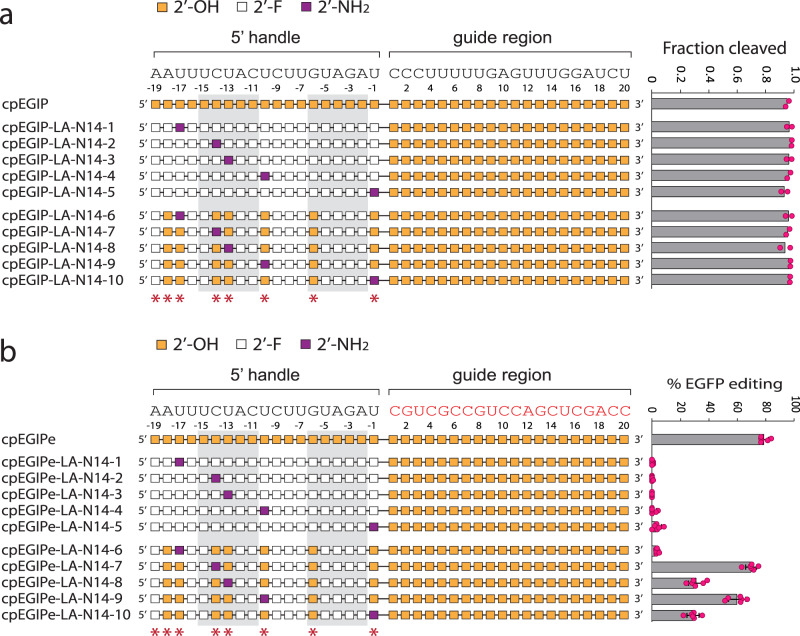
In vitro cleavage and cell-based editing activity of *As*Cas12a using crRNA guides modified with 2′-F and 2′-NH_2_ combinations. Chemical modification patterns are illustrated with corresponding mean in vitro cleavage activity (*n* = 2) (**a**) or mean cell-based editing activity (*n* = 6) (**b**) shown to the right. Error bars are standard deviation and replicates are biological. For both panels, nucleotides of the 5′ handle that are predicted from structural data to make polar contacts through 2′-OH hydrogen bonding are indicated with red asterisks below. The base-paired stem nucleotides of the 5′ handle pseudoknot are enclosed with light gray boxes. In vitro cleavage and cell-based editing assays utilize the distinct guide sequences that are shown above each schematic. Source data are provided as a Source Data file.

The positive performance of 2′-F but generally poor performance of 2′-araOH and OxN indicated that smaller structural perturbations of the ribose and the ability to provide hydrogen-bonding potential at the 2′ position were desirable. To determine if further RNA reduction at putative critical 2′-OH positions might be possible, we identified 2′-amino (2′-NH_2_) as a potentially promising modification. The relatively small amino moiety and its ability to both donate and accept hydrogen bonds made it an attractive replacement for RNA. In addition, the pKa of the 2′-amino should be 6.2, as determined by Eckstein and colleagues^96^, making the major species non-protonated at physiological pH. 2′-NH_2_ nucleotides have been used for other technologies, like aptamer and ribozyme development^97–99^, but have not been applied to CRISPR guide RNA modification previously. Starting with an all-2′-F pseudoknot, we sought to replace the putative critical positions individually with 2′-NH_2_ to ensure that in vitro activity was not affected. Because 2′-NH_2_ is an uncommon modification and only pyrimidine monomers were commercially available, we did not substitute positions -18 and -6. All the crRNAs containing a 2′-NH_2_ substitution in the 2′-F pseudoknot (cpEGIP-LA-N14-1 through -LA-N14-5) were highly active in vitro (Fig. 4a), indicating no negative impact on intrinsic enzyme activity. However, none of these designs targeting EGFP in cells showed significant gene editing activity (Fig. 4b).

To begin with modified pseudoknots more likely to generate gene editing activity, we created the equivalent of cpEGIP-SJ14 (~80% editing) but walked a 2′-NH_2_ through each remaining native RNA nucleotide position (except -18 and -6). These new designs, cpEGIP-LA-N14-6 through -LA-N14-10, all exhibited robust in vitro cleavage activity (Fig. 4a). When targeting the stably expressed EGFP gene in cells, these designs presented varying degrees of gene editing. Substitution at position -17 with 2′-NH_2_ abrogated gene editing activity (cpEGIPe-LA-N14-6). Substitution at positions -13 and -1 resulted in approximately 30% gene editing (cpEGIPe-LA-N14-8 and -LA-N14-10). In contrast, substitutions with 2′-NH_2_ at positions -10 and -14 (cpEGIPe-LA-N14-9 and -LA-N14-7) provided ~60–70% editing, which is substantial considering that the native RNA pseudoknot typically provides 80% editing. Interestingly, substitution at position -14 resulted in similar activities whether 2′-F (cpEGIPe-SJ14-3) or 2′-NH_2_ (cpEGIPe-LA-N14-7) were used, suggesting relatively flexible accommodation at this position. 2′-NH_2_ was not suitable for replacing RNA at positions -17 or -1, which was also the case with 2′-F (cpEGIPe-SJ14-2 and cpEGIPe-SJ14-7). However, position -13 was differentially affected, with 2′-F being very well-tolerated (cpEGIPe-SJ14-4), whereas 2′-NH_2_ was poorly tolerated (cpEGIPe-LA-N14-8). Amines do not form hydrogen bonds as stably as alcohols and are bulkier at the 2′ position than OH and F, which may explain differential activity effects.

### *Trans* cleavage activity of modified Cas12a crRNAs

**Fig. 5:**
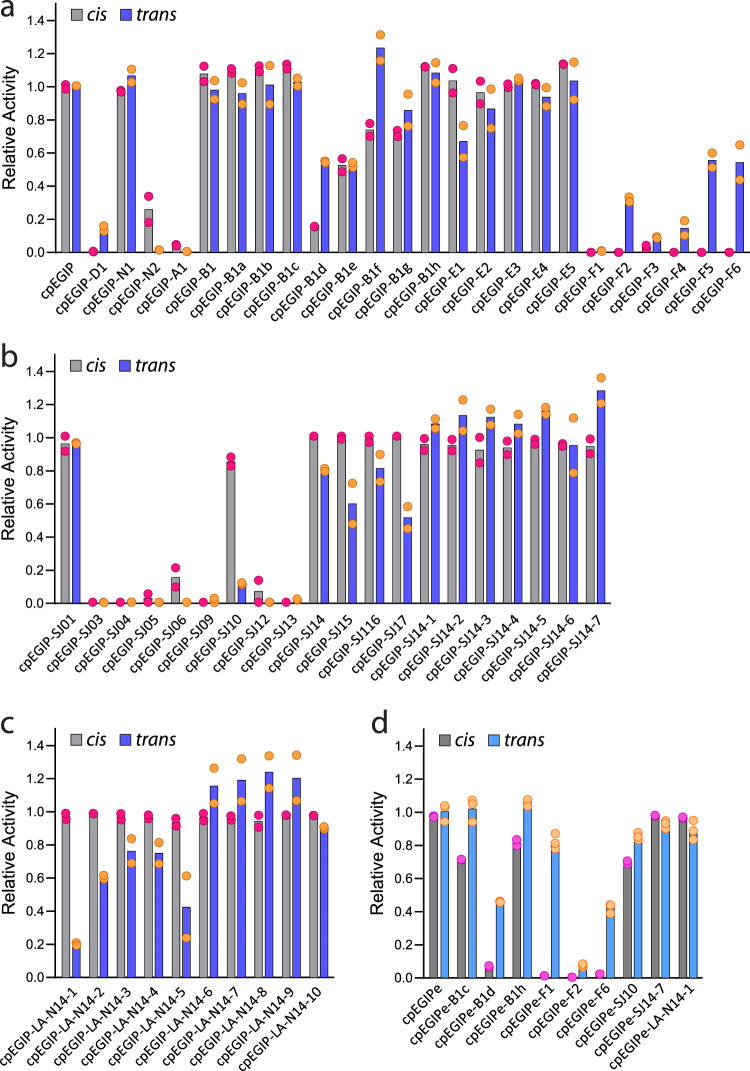
Chemical modification of the *As*Cas12a crRNA 5′ handle modulates non-sequence-specific *trans* ssDNase activity. **a**–**d** Comparing dsDNA sequence-specific *cis* and ssDNA non-specific *trans* activity of *As*Cas12a with crRNAs containing modified 5′ handles. Error bars are standard deviation and replicates are biological. The guide sequence used in panels (**a**–**c**) (cpEGIP) is designed to target an EGFP-containing plasmid for in vitro *cis* cleavage while the guide used in panel (**d**) (cpEGIPe) is designed to target a chromosomally integrated EGFP gene for cell-based editing. For all samples, two separate replicates were performed (*n* = 2). Source data are provided as a Source Data file.

Differences in sequence-specific *cis* cleavage in vitro versus cell-based gene editing prompted us to investigate whether chemically modified 5′ pseudoknots impacted non-sequence-specific *trans* cleavage activity. We employed a fluorescence-based assay similar to that originally reported by Chen and coworkers^41^ (Supplementary Fig. 3a). We systematically screened all crRNAs with modified pseudoknots and plotted their maximum *trans* activity after 1 h of reaction time. We found that levels of *trans* and *cis* activity closely mirrored one another for most crRNAs (Fig. 5). This would be expected since *trans* cleavage activity relies on the same active site as *cis* cleavage activity^45,46^. However, a few crRNAs with modified 5′ pseudoknots generated differential *cis* versus *trans* activity. For example, cpEGIP-B1d and cpEGIP-F6 both had little or no *cis* activity but exhibited about 50% *trans* activity (Fig. 5a). However, after incubation for 2 h, both reached similar activity levels as the all-RNA control crRNA (Supplementary Fig. 3b). In contrast, cpEGIP-SJ10 and cpEGIP-LA-N14-1 both exhibited very high *cis* cleavage but low *trans* cleavage activity (~10%) (Fig. 5b, c). Incubation for over 2 h resulted in no additional *trans* cleavage for cpEGIP-SJ10 and less than 40% for -LA-N14-1 (Supplementary Fig. 3b).

To determine whether the apparent differential *cis*–*trans* activity was guide-sequence dependent, we ligated the modified 5′ pseudoknot to the cpEGIPe guide sequence used for editing in cells. With this guide sequence, both cpEGIPe-B1d and cpEGIPe-F6 still produced markedly low *cis* but about 50% *trans* activity (Fig. 5d), which reached full *trans* activity after an additional 60 min of incubation (Supplementary Fig. 3c). In addition, cpEGIPe-F1 still produced almost no *cis* activity but now generated very high *trans* activity. Two modified crRNAs that had previously shown high *cis* but low *trans* activity, cpEGIPe-SJ10 and cpEGIPe-LA-N14-1, now exhibited similar *cis*–*trans* activity profiles. One possible explanation for these results is that chemical modifications may enable conformational changes to Cas12a that allow *trans* cleavage without target binding. However, *trans* cleavage assays lacking target DNA for several crRNAs did not produce any significant *trans* activity (Supplementary Fig. 4a). An alternative explanation for *trans* activity with little or no *cis* activity could potentially be “nicking” of one of the target DNA duplex strands. This could conceivably activate Cas12a *trans* activity but not be readily observed in typical *cis* cleavage assays, which use non-denaturing gel electrophoresis. Therefore, we individually 5′ radiolabeled non-target (sense) and target (antisense) strands and performed *trans* cleavage assays on duplexes using cpEGIPe and cpEGIPe-B1d, -F1, -F6, and -SJ14-7 crRNAs, followed by resolution of reaction products on denaturing polyacrylamide gels (Supplementary Fig. 4b). The cleavage of individual duplex strands very closely matched that of our previous *cis* assays, indicating that differential strand nicking was not a likely explanation for high *trans* but low *cis* activity. To further investigate the mechanism of apparent differential *cis*–*trans* activity, we generated hybrid dsDNA targets that incorporated a partially PS-modified non-target (sense) or target (antisense) strand (Supplementary Fig. 5a)^45^. The PS-modified target strand was observed to consistently reduce *trans* activity by about 60% for both native and chemically modified pseudoknot-containing crRNAs while a PS-modified non-target strand had little or no effect (Supplementary Fig. 5b). These results are consistent with *trans* activity first requiring *cis* cleavage of the target strand^45^. Thus, we conclude that high catalytic turnover of *trans* activity^41^ versus the low turnover of *cis* activity is the most likely explanation for apparent differential *cis*–*trans* activity. The modified crRNAs that displayed differential *cis*–*trans* activity likely fell into an activity regime that made this effect easily observable. Nonetheless, chimeric RNA–DNA pseudoknots did not appear to change the intrinsic enzyme mechanism of *trans* cleavage (Supplementary Fig. 5b).

**Fig. 6:**
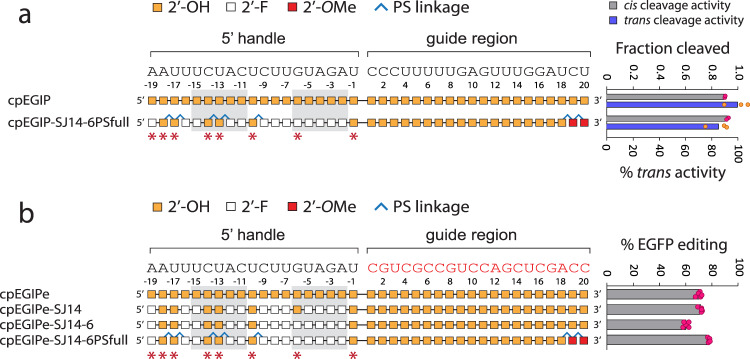
In vitro *cis* and *trans* cleavage activity and cell-based editing of *As*Cas12a using a heavily modified pseudoknot crRNA guide. Chemical modification patterns are illustrated with corresponding mean in vitro *cis* and *trans* activity (*n* = 2) (**a**) or mean cell-based editing (*n* = 5) (**b**) shown to the right. Error bars are standard deviation and replicates are biological. Nucleotides of the 5′ handle that are predicted from structural data to make polar contacts through 2′-OH hydrogen bonding are indicated with red asterisks below. The base-paired stem nucleotides of the 5′ handle pseudoknot are enclosed with light gray boxes. In vitro cleavage and cell-based editing assays utilize the distinct guide sequences that are shown above each schematic. Source data are provided as a Source Data file.

The crRNA possessing the maximum number of modifications and exhibiting little or no loss in editing was cpEGIPe-SJ14-6. However, this modification scheme retained six RNA residues, which may be subject to RNase attack in therapeutic contexts. Thus, to further stabilize these final positions, we replaced the phosphodiester linkage 3′ to each remaining RNA residue with PS linkages. This newly modified pseudoknot was ligated to the cpEGIPe guide possessing two terminal 3′ PS linkages and 2′-*O*-methyl nucleotides to further stabilize against 3′ exonucleases. This new modified crRNA, cpEGIPe-SJ14-6PSfull, exhibited full cis, trans and editing activities (Fig. 6). Thus, *As*Cas12a crRNAs can be generated with heavily modified pseudoknots that should protect every nucleotide from nucleases and retain robust editing activity. To test the nuclease resistance of several pseudoknots and ligated crRNAs, we also performed serum stability assays (Supplementary Fig. 6a). Native crRNA (cpEGIPe) and an RNA–DNA chimeric pseudoknot (cpEGIPe-F6) were completely digested under the conditions tested. The cpEGIPe-SJ01 pseudoknot, composed entirely of 2′-F, was preserved while the cpEGIPe-SJ14 pseudoknot, containing 2′-F and seven RNA residues, was significantly degraded. To test the effect of additional PS modifications, full-length crRNAs were generated by enzymatic ligation. Addition of two terminal PS modifications to the SJ14-6 design (cpEGIPe-SJ14-6PS) improved stability of the modified 5′-pseudoknot slightly (lanes 8 and 10). In contrast, the 5′ pseudoknot of cpEGIP-SJ14-6PSfull was well preserved against degradation (lane 12). These results confirm that select 2′-F and PS modification of the 5′ pseudoknot can stabilize against common serum nucleases.

To determine whether pseudoknot modifications may be impacting enzyme activity through large conformational effects, we performed limited trypsin hydrolysis on *As*Cas12a RNP complexes (Supplementary Fig. 6b). *As*Cas12a by itself, assembled as a native RNP with cpEGIPe, or further bound to its cognate dsDNA target revealed unique limited trypsin digestion patterns. However, no difference in the trypsin digestion pattern was observed when comparing native RNP complexes with RNPs assembled from heavily modified pseudoknot guides (cpEGIPe-SJ14-6 and cpEGIPe-SJ14-6PSfull) previously shown to possess robust in vitro cleavage and editing activity (Figs. 3 and  6). Further addition of a cognate dsDNA target to modified RNP complexes also showed no differences when compared to the native RNP complex with bound target. Thus, crRNAs with modified pseudoknots that confer high editing activity do not perturb gross *As*Cas12a conformation.

## Discussion

Chemical modification of RNA is a method to probe the role of functional groups in RNA and RNP structure and biochemical activity^71,100^. It is also a critical step in converting RNA into drug-like molecules^66^. In this study, we have used chemical modification to investigate the necessity of the ribose 2′-OH group in the *As*Cas12a crRNA 5′ pseudoknot structure for enzyme activity. The primary question was whether the pseudoknot structure was too complex or sensitive for efficient chemical modification, which previous studies had suggested^74,78,81^. The rationale for our approach is the high likelihood that therapeutic applications of Cas12a that require direct delivery of the crRNA will also require heavy or complete chemical modification, as is the case for siRNAs. In particular, the lability of RNA is directly linked to the presence of a 2′-OH that can help catalyze intramolecular phosphodiester bond self-cleavage or attract ribonucleases^101^. The 5′ pseudoknot anchors crRNA binding to Cas12a and is highly conserved across the CRISPR-Cas12a family^16,39,102^. The conservation of pseudoknot sequence and structure suggests that the 5′ pseudoknot architecture is fine-tuned for Cas12a function and cannot be easily perturbed^16,102^. However, if successful chemical modification schemes can be identified, they should translate to other Cas12a proteins if RNA-protein contacts are preserved, which appears to be the case from recent structural investigations^20,39,103^.

Here we focused primarily on 2′-deoxyribose, or DNA, and 2′-F as substitutions and also included modifications designed to potentially retain hydrogen bonding between pseudoknot nucleotides or with *As*Cas12a protein. We have demonstrated that as many as 13 out of 19 nucleotides can be converted to 2′-F with no impact on efficiency of EGFP editing in human cells. Attempting to systematically modify remaining putative critical 2′-OH positions with 2′-F, OxN, 2′-araOH, and 2′-NH_2_ resulted in moderate success. However, it was clear that a few positions are resistant to modification and seem to rely heavily on the 2′-OH group. For example, our results suggested that the more centrally positioned 2′-OH contacts, especially at residues -14, -10, and -6, were more tolerant to substitution while terminal residues (-18, -17, and -1) were less tolerant. Although OxN and 2′-araOH were not sufficient substitutes for ribose, it appears that a few 2′-NH_2_ and 2′-deoxy could be incorporated in the context of 2′-F. Positions most amenable to these modifications are position -19 and more central positions like -6. In summary, removal of all but a handful of ribose sugars is achievable in the 5′ pseudoknot structure of *As*Cas12a crRNAs while still maintaining very high gene editing activity in cells. In addition, selective placement of PS linkages 3′ to remaining RNA nucleotides further stabilized the RNA while not compromising editing activity. A similar approach succeeded in stabilizing remaining RNA positions in a Cas9 guide RNA, although editing in the final heavily modified RNA guide was significantly reduced^70^. Previously, full PS modification of the native 5′ pseudoknot of the *As*Cas12a crRNA resulted in a substantial loss of editing in cells^81^.

Previous attempts to modify the 5′ pseudoknot sequence replaced entire blocks of contiguous nucleotides, several at a time, with DNA, 2′-F, or 2′-*O*-methyl^74,78^. This approach is typically suitable for oligonucleotides that function almost exclusively through Watson–Crick base-pairing, such as ASOs or siRNAs^66,104^. However, it resulted in no editing activity, likely due to loss of critical 2′-OH contacts. Overall, these results reinforce the need to incorporate molecular and structural data into modification design and the value of nucleotide analogs that can mimic or substitute the properties of native ribose.

Ultimately, complete ribose chemical modification of the entire crRNA would be desirable for precise control over therapeutic development. Attempts to modify the guide region of Cas12a crRNAs explored 2′-*O*-methyl, 2′-F, DNA, LNA, UNA, and PS linkages^74,81^. The general conclusion from these studies was that modification at or near the 3′ end of the guide region is relatively well-tolerated for most modifications. However, modification at the 5′ end of the guide, and therefore within or near the “seed” region, were very poorly tolerated. A notable feature of the seed region of both Cas12a and Cas9 CRISPR systems is the concentrated presence of conserved 2′-OH contacts with the Cas protein^12,20^. In addition, the ability for Cas9 to recognize and bind DNA targets, as well as catalyze cleavage, has been shown to be dependent on the formation of an A-form-like duplex between the guide and target DNA^69,71^. Together, the 2′-OH and A-form-like architectural requirements of crRNAs reflect on the unique co-evolution of bacterial CRISPR-Cas systems to use RNA as guides^14,105,106^. Complete chemical modification of guide RNAs, such as for therapeutic CRISPR-based editing, will require addressing the 2′-OH bottleneck that has finally become apparent for Cas9 and Cas12a, and probably for other Cas enzymes. Nonetheless, our results combined with previous studies now provide a roadmap to near-complete chemical modification across the entire Cas12a crRNA. We propose that a primarily 2′-F and 2′-*O*-methyl oligonucleotide, with some DNA or 2′-NH_2_ nucleotides, combined with selective PS modification, may be sufficient to provide drug-like profiles with favorable editing activity. Exploring a greater diversity of modifications at well-placed positions may further unlock efficient editing with little or no native RNA residues. In addition, other properties of Cas12a will need to be investigated, such as the impact of chemical modification schemes on off-target editing, as heavier modification is incorporated.

A recurring theme in crRNA chemical modification studies, especially with Cas9, is that heavy modification schemes successful for in vitro cleavage often do not translate into high editing efficiency in cells^61,70,71,80^. For example, all RNA nucleotides can be replaced with 2′-F in the Cas9 crRNA^71^, and now the Cas12a pseudoknot, and provide very high cleavage in vitro. However, these crRNAs provide little or no editing in cells. Converting predicted 2′-OH polar contact positions back to RNA usually rescues gene editing^70,71^. Thus, it appears that intrinsic biochemical activity is not dependent on 2′-OH contacts in bulk reactions in vitro but these become critical within cells, perhaps due to low biochemical RNP concentrations, more complex chromosomal DNA targets, or other unexplored biochemical or cellular factors^107^. Experiments like precision biochemical, thermodynamic, and structural studies, as well as sophisticated single-molecule or gene editing assays in cells, may be necessary to fully understand this phenomenon and unlock “RNA-free” CRISPR-Cas editing for advanced therapeutic control.

The discovery of non-sequence-specific ssDNase cleavage, or *trans* activity, by Cas12a was quickly converted into an amplification-based diagnostic method^41^. After sequence-specific binding and cutting (*cis* cleavage) of a DNA target, the catalytic domain of Cas12a is proposed to become solvent exposed such that ssDNA is accessible to the active site^45,46^. This results in high turnover degradation of ssDNA. Once DNA target paired to the guide RNA is released, ssDNase cleavage activity should cease as Cas12a returns to a pre-catalytic structural state^46^. Thus, *trans* cleavage activity of Cas12a appears to be intrinsically tied to its *cis* cleavage activity, turnover of *cis* cleavage products, and conformational-state transitions.

In this study, we initially found what appeared to be differences in *cis* versus *trans* cleavage activity for a few crRNAs possessing chemically modified pseudoknots. Certain modification patterns or schemes contributed to this effect. Ultimately, the differential *cis*–*trans* activity observed here appears to be a consequence of variable *cis* cleavage compared to catalytic *trans* turnover rates for certain guides. This can result in variable ratios of *cis* to *trans* activity at time points within a certain activity regime. However, *trans* activity by modified crRNAs still required cleavage of the target antisense DNA strand in a dsDNA target. Thus, the overall mechanism of trans activity was unaffected. The pseudoknot is proposed to influence catalysis by modulation of *As*Cas12a conformational transitions^45,46^, which may explain how various activity profiles were achieved by modified pseudoknots. If differential *cis*–*trans* cleavage activity could be controlled predictably, it might prove useful for some applications. For example, binding to a specific on-target DNA without cleaving (*cis* activity) but still allowing *trans* activity, could provide *trans* activity for long durations and possibly improve diagnostics. Conversely, *cis* cleavage without activation of *trans* cleavage might improve the specificity or safety of other applications, like gene editing^108^. Recently, it was reported that extending the 5′ or 3′ ends of the LbCas12a crRNA could increase on-target activity, and especially enhance *trans* cleavage rates^77^. Thus, multiple types of crRNA modification may be useful for modulating *cis* specificity and *trans* turnover.

## Methods

### RNA synthesis

DNA oligonucleotides, crRNAs, and DNA–RNA chimeric 5′-handle RNAs were synthesized and purified by Integrated DNA Technologies (IDT). Full-length crRNAs, including DNA–RNA and chemically modified crRNA, were ligated using splint ligation (Supplementary Fig. 1 and Supplementary Tables 1 and 2). Chemically modified 5′-handles were custom synthesized and mass confirmed by mass spectrometry (Supplementary Table 2). Custom, chemically synthesized oligonucleotides may be available upon request and after appropriate inter-institutional material transfer agreements are approved. Single-stranded fluorophore-quencher (FQ) DNA substrate was synthesized commercially by IDT.

Oligonucleotide chemical syntheses were carried out using either an ABI 3400 DNA synthesizer (Applied Biosystems) or a MerMade 12 Oligonucleotide synthesizer (BioAutomation) on a Unylinker CPG (ChemGenes) solid support at a 1 µmol scale. Conventional 2′-tert-butyl-dimethylsilyl (TBDMS) ribonucleoside, 2′-fluoro-ribonucleoside (2′-FRNA), and 2′-trifluoroacetyl amino phosphoramidites (ChemGenes) were used, along with newly synthesized oxepane phosphoramidites. Phosphoramidites were dissolved in MeCN (0.15 M) and activated with 5-ethylthio-1H-tetrazole (0.25 M in MeCN). Capping of failed couplings was carried out by the simultaneous delivery of acetic anhydride in pyridine/THF and N-methylimidazole (16% in THF) and contacting the solid support for 6 s. Oxidation of the phosphite triester intermediates was affected with 0.1 M iodine in pyridine/H_2_O/THF (20 s). A solution of 3% trichloroacetic acid in THF, delivered over 95 s, was used to deprotect DMTr groups.

Deprotection and cleavage of oligonucleotides from the solid support was achieved by treatment with 1 mL of cold 29% aqueous ammonia/ethanol (3:1, v/v) for 16 h at 65 °C (or 48 h at room temperature for oxepane-containing oligonucleotides). The samples were centrifuged and the supernatant was transferred to a clean 1.5 mL microcentrifuge tube and vented for 30 min, chilled on dry ice, and evaporated to dryness. Removal of the 2′-TBDMS protecting groups for the RNA-containing oligonucleotides was achieved by treatment with a 300 µL solution of NMP/Et_3_N/TREAT-HF (3:4:6, v/v) for 90 min at 65 °C (samples in the SJ series were desilylated by exposure to neat TREAT-HF for 48 h), followed by quenching with 3 M NaOAc buffer (50 µL; pH 5.5) and precipitation of the crude oligonucleotide from cold butanol (1 mL, −20 °C). Samples were chilled on dry ice for 30 min and then centrifuged. After removing the supernatant, the remaining pellet was evaporated to dryness, taken up in autoclaved Milli-Q water (1 mL), and filtered.

Crude oligonucleotides were purified by ion exchange (IE) HPLC. cpEGIP-LA oligonucleotides were purified on a Waters 1525 instrument using a Protein-Pak DEAE 5PW anion exchange column (21.5 mm × 150 mm). A buffer system consisting of buffer A (10 mM NaOAc, 20% MeCN in Milli-Q water) and buffer B (0.5 M LiClO_4_, 10 mM NaOAc, 20% MeCN in Milli-Q water) was used for analysis and purification. Using a gradient of 0–50% LiClO_4_ (buffer B) over 50 min (10 mL/min, 60 °C), the desired compounds eluted at around 30 min. cpEGIP-SJ oligonucleotides were purified on an Agilent 1200 Series Instrument using a Protein-Pak DEAE 5PW column (7.5 × 75 mm) at a flow rate of 1 mL/min, with a buffer system consisting of buffer A (Milli-Q water) and buffer B (1 M LiClO_4_ in Milli-Q water) using a method of 0–60% buffer B over 37 min at 60 °C. Following collection of the desired peaks, excess LiClO_4_ salts were removed using Gel Pak 2.5 size exclusion columns (Glen Research). Purified oligonucleotides were characterized by electrospray ionization-mass spectrometry (Supplementary Table 2) and quantitated by UV spectroscopy. Extinction coefficients were determined using the IDT OligoAnalyzer tool (https://www.idtdna.com/calc/analyzer). Extinction coefficients for RNA were used for oligonucleotides containing oxepane modifications.

### crRNA splint ligation

crRNA guide sequence (200 pmol), synthesized with a 5′ phosphate, was ligated to 200 pmol of 5′-handle RNA (synthesized with a 3′-OH) using T4 DNA ligase^87^ (Supplementary Table 1) For DNA ligase, 220 pmol splint DNA, complementary to both guide and 5′-handle RNA, was annealed to 200 pmol of each crRNA portion in RNA resuspension buffer (5 mM Tris, pH 7.4, 0.5 mM EDTA) at 65 °C, then slow-cooled to room temperature. Ligation was performed using 1 µL of concentrated DNA ligase (30 U/μL) (Thermo Scientific, EL0013), 1x ligation buffer (400 mM Tris-HCL, 100 mM MgCl_2_, 100 mM DTT, 5 mM ATP), and 0.5 µL of SUPERase-In (Invitrogen) in a final volume of 100 μL. The reaction was incubated for 90 min at 37 °C and stopped by adding 1 µL of 0.5 M EDTA pH 8 and phenol-chloroform extracted. Ligation products were visualized by resolving on 15% denaturing polyacrylamide gels and staining with methylene blue. Full-length ligation products were gel-purified by crush-and-soak elution, phenol-chloroform extracted, ethanol precipitated, and quantified by measuring absorbance at 260 nm and calculated extinction coefficients using nearest neighbor approximations with Beer’s Law. Electrospray ionization-mass spectrometry was used to confirm the mass of a few full-length, ligated crRNAs to ensure ligation and gel-purification was proceeding properly.

### *As*Cas12a enzyme expression and purification

Plasmid encoding of an *As*Cas12a was obtained from Addgene (79007). *As*Cas12a proteins were prepared similarly to that previously described for *Sp*Cas9^109^. Briefly, protein expression was induced in Rosetta (DE3) cells grown in Luria-Bertani (LB) broth with 0.2 mM isopropyl thiogalactopyranoside (IPTG) at 18 °C for 16 h. Cell pellets were resuspended in 6 mL chilled binding buffer (20 mM Tris-HCl, pH 8.0, 250 mM NaCl, 1 mM PMSF, 5 mM imidazole) per 0.5 L of culture. Resuspended cells were sonicated and clarified by centrifugation. For the 0.5 L of culture, 5 mL His-Pur Cobalt-CMA resin (Thermo Scientific) was equilibrated with binding buffer and the supernatant added to the equilibrated resin and incubated at 4 °C for 1 h with inversion to mix every 15 min. The column was washed sequentially with at least 10 bed volumes of increasing concentrations of NaCl in wash buffer (20 mM Tris-HCl, pH 8.0, 10 mM imidazole, 0.25/0.5/0.75/1.0 M NaCl). Protein was eluted with 130 mM imidazole buffer (20 mM Tris-HCl, pH 8.0, 250 mM NaCl, 200 mM imidazole). Purified *As*Cas12a enzyme was concentrated and buffer exchanged using centrifugal concentrators (Sartorius, 30,000 MWCO) into 2x storage buffer (40 mM Tris, pH 7.5, 300 mM KCl, 1 mM EDTA, and 2 mM DTT). Then one volume of glycerol added to obtain a final of 50% glycerol. Protein stocks were then stored at −80 °C. Concentration of *As*Cas12a was determined by UV absorbance at 280 nm using a calculated extinction coefficient and Beer’s law.

### In vitro *As*Cas12a *Cis* cleavage activity assays

A 1 kb fragment of the target EGFP gene was PCR-amplified using “cpEGIP_vitro targ” primers (Supplementary Table 1) from plasmid DNA (Addgene, 26777) and purified by phenol-chloroform extraction and ethanol precipitation. Target DNA (185 ng) was spotted into tubes and combined with the *As*Cas12a (0.75 µM final) and crRNA (0.3 µM final) in 1x cleavage buffer (20 mM Tris-HCl, pH 7.5, 100 mM KCl, 5% glycerol, 1 mM DTT, 0.5 mM EDTA, 2 mM MgCl_2_) supplemented with 0.1 mg/mL of purified yeast tRNA. Molar excess of the *As*Cas12a over crRNA allows RNP concentration approximation by using crRNA concentration. Although a fraction of the Cas enzyme remains unbound, we have previously found this approach to be an accurate method for predicting the concentration of actual assembled RNP complexes^69,71^. The 40 µL reaction was incubated at 37 °C for 2 h. The reaction was then treated with 10 µg of RNase A (Thermo Scientific) for 15 min followed by 20 µg of Proteinase K (Thermo Scientific) for 15 min at room temperature. The reaction products were then precipitated in 10 volumes of 2% LiClO_4_ in acetone for >1 h at −20 °C. Precipitated reactions were centrifuged and washed with acetone, air dried, and resuspended in gel-loading dye (10% glycerol, 1x TBE, orange G dye) and resolved on 1.5% agarose gels. Agarose gels were stained with ethidium bromide and visualized using a UV imager. The fractions of target cleaved versus uncleaved were quantified using ImageJ software (v1.43u). Dot plot data points represent experimental replicates, not technical replicates.

For cleavage time-course assays, *As*Cas12a and crRNA (cpEGIP or cpEGIP-SJ14) were assembled at room temperature for 10 min. The RNP was then added to a target DNA in at 37 °C in a final of 1x cleavage buffer and reactions stopped at indicated time points by precipitation with 2% LiClO_4_ in acetone and incubated at −20 °C. Samples were pelleted by centrifugation then washed with acetone. After samples were air dried, they were resuspended in 1x loading dye (10% glycerol, 1x TBE, orange G dye), treated with RNase A and proteinase K as described above, and cleavage products resolved on 1.5% agarose gels. Quantification was performed as described above.

### Generation of HEK293T cells stably expressing EGFP and *As*Cas12a

HEK293T cells stably expressing EGFP were a kind gift from Dr. Wen Xue^61^. We subcloned *As*Cas12a from pY010 (pcDNA3.1-hAsCpf1) (Addgene, 69982) into pLJM1-EGFP lentivector^110^ (Addgene, 19319) such that the EGFP gene was replaced with *As*Cas12a. Subcloning was performed with *Nhe*I and *Eco*RI restriction enzymes and resulted in retention of the C-terminal NLS and HA tag on *As*Cpf1 in the new vector, pLJM1-*As*Cas12a (Supplementary Fig. 2a). The PGK-driven puromycin resistance gene was replaced with a hygromycin resistance gene downstream of an internal ribosomal entry site. HEK293T cells were transfected with pLJM1-*As*Cas12a, pCMV-VSVG (Addgene, 8454), and pCMV-dR8.2 dvpr (Addgene, 8455)^111^ using the calcium phosphate technique. Medium was replaced 18 h after transfection. Lentiviral containing medium was collected 48 h later and centrifuged at 1000*g* for 5 min. Hexadimethrine bromide was added to the medium (8 µg/mL) and the medium was used to infect HEK293T-EGFP cells on 6-well plates for 8 h.

Transfection of cells with pLJM1-*As*Cas12a and staining with anti-HA (Santa Cruz Biotechnology, 1:1000) revealed primarily cytoplasmic localization (Supplementary Fig. 2b). Therefore, we cloned an additional 3x NLS tag onto the N-terminus of *As*Cas12a to generate pLJM1-3xNLS-*As*Cas12a. Staining with anti-HA revealed exclusively nuclear staining (Supplementary Fig. 2b). Stably integrated cells were selected using 100 µg/mL hygromycin and a clonal cell line was expanded. Expression and nuclear localization of *As*Cas12a was confirmed in the stable cell line (Supplementary Fig. 3c). Stable pLJM1-3xNLS-*As*Cas12a HEK293T-EGFP cells may be available upon request and after appropriate inter-institutional material transfer agreements are approved.

### Cell-based editing measured by flow cytometry

HEK293T cells expressing EGFP and *As*Cas12a were grown in Dulbecco’s modified eagle’s medium (DMEM) with 1x non-essential amino acids (NEAA), 5% cosmic calf serum (CCS) and 2.5% fetal bovine serum (FBS) without antibiotics. Cells were reverse-transfected (40,000 cells) in six experimental replicates in 96-well plates with 20 pmol of crRNA and 0.3 µL RNAiMAX lipid (Invitrogen) in a final reaction of 200 μL of OptiMEM. After 8 h, one volume of media containing 5% FBS and 5% CCS was added to cells and further incubated overnight. Media was then replaced with full media and cells grown for an additional 4 days. For flow cytometry, cells were washed with 200 μL phosphate-buffered saline (PBS) and trypsinized by adding 70 μL of trypsin-EDTA solution. Then, 100 μL of media was added to the cells. The cells were spun for 5 min at 300*g* at room temperature. Cells were washed again with 200 μL PBS, resuspended in 200 μL PBS and counted in an Attune flow cytometer. EGFP was detected using the blue laser (BL1 channel). At least 20,000 events were collected and analyzed by Attune software (v3.12). The cells were gated based on forward and side scattering (FSC-A/SSC-A) to remove cell debris, gated to select single cells, and gated to select EGFP-positive cells. The quadrant gate was established using the signal from non-EGFP-expressing control cells. Untreated HEK293T cells expressing EGFP and *As*Cas12a contained ∼5% non-fluorescent cells (Fig. S7). The average from five or six replicates (see figure legends) was used for background subtraction to determine the extent of cell-based editing after treatment. Two crRNAs with different guide sequences, cpEGIP and cpEGIPe, were initially screened. cpEGIPe provided substantial editing activity (75–80%) and was chosen for subsequent modification and editing experiments in cells (Supplementary Fig. 2d).

### Fluorophore-quencher (FQ) *Trans* cleavage reporter assay

*As*Cas12a and crRNA were preassembled into RNP complex by incubating 500 nM *As*Cas12a with 550 nM crRNA and 25 nM DNA target in 1X cleavage buffer (20 mM Tris-HCl, pH 7.5, 100 mM KCl, 5% glycerol, 1 mM DTT, 0.5 mM EDTA, 2 mM MgCl_2_) on ice for 15 min. Certain experiments either omitted target DNA, crRNA, or used PS-modified target at the same concentrations as indicated above. Reactions were initiated by adding FQ ssDNA substrate to a final of 3 µM and placing the plate in a Bio-Rad CFX96 instrument with the block set at 4 °C. The block was cycled to 37 °C and the plate was read using the SYBR-only channel every 53 s (reading takes 7 s) such that fluorescence readings were collected at 1 min intervals. Fluorescence was collected for up to 150 min. Maximum fluorescence values at 1 h were used to compare non-sequence-specific *trans* cleavage activity of varying crRNAs containing modified 5′ pseudoknots. Error was calculated by standard deviation, representing three or more experimental replicates.

For strand-nicking cleavage assays utilizing radiolabeled target DNA strands, individual DNA strands (cpEGIPe target sequence) were 5′ radiolabeled with T4 polynucleotide kinase and ^32^P-γ-ATP as previously described^112^. Radiolabeled DNA strands were then gel-purified from a 15% denaturing Urea-PAGE by crush-and-soak elution after visualizing radioactive bands by autoradiography. Duplexes with differentially radiolabeled sense (non-target) or antisense (target) strands were annealed and slow-cooled with a ~1.2-fold molar excess of unlabeled complementary strand. Reaction conditions then utilized those of a standard *trans* cleavage assay but lacking a FQ-labeled ssDNA substrate. Reactions proceeded for 1 h before being stopped by phenol-chloroform extraction. Cleavage products were resolved on a 15% denaturing PAGE and exposed to phosphorimager for visualization.

### Limited trypsin hydrolysis of *As*Cas12a protein and RNP

Trypsin proteolysis was performed using 30 μg of *As*Cas12a in the presence and absence of crRNA and double-stranded target DNA (1:1.5:2 molar ratio)^113^. The RNP was incubated at room temperature for 5 min. The resulting samples were incubated with Trypsin-EDTA solution (0.05%, Invitrogen) at a mass ratio of 100:1 and the partial proteolysis was conducted at 37 °C for 15 min. The reaction was stopped by the addition of SDS-PAGE loading buffer and heating the samples for 5 min at 95 °C. The reaction products were analyzed by 12% SDS-PAGE and stained with Coomassie brilliant blue G-250 in 50% (v/v) methanol and 10% (v/v) acetic acid then destained in the same solution without dye.

### Serum stability of modified Cas12a pseudoknots and crRNAs

Serum stability test was performed using 300 pmol of pseudoknot or crRNA and 5 µL of 10% FBS in 1x PBS in a total reaction volume of 50 µL. Reactions were incubated at 37 °C and then stopped after 2 h by adding 1 µL of 0.5 M EDTA. RNA was extracted by phenol-chloroform extraction, precipitated with 2% LiClO_4_, and resolved on a 15% TBE-buffered denaturing (7 M urea) polyacrylamide gel. Gels were stained with methylene blue to visualize digestion patterns.

### Reporting summary

Further information on research design is available in the Nature Research Reporting Summary linked to this article.

## Supplementary information

Supplementary information.

Reporting summary.

## Data Availability

Any additional data or materials can be obtained upon reasonable request. Source data are provided with this paper.

## Source data

Source Data file.
