# Supplementary material for: Gene editing with CRISPR-Cas12a guides possessing ribose-modified pseudoknot handles
Source: Nat Commun. 2021 Nov 15;12:6591. doi: 10.1038/s41467-021-26989-z (PMC8593028; doi:10.1038/s41467-021-26989-z)
Supplement: Supplementary file 1 — Supplementary information. [file 41467_2021_26989_MOESM1_ESM.pdf]

SUPPLEMENTARY DATA

Gene Editing with CRISPR-Cas12a Guides Possessing Ribose-Modified Pseudoknot Handles

Eman A. Ageely[1], Ramadevi Chilamkurthy[2], Sunit Jana[3,*], Leonora Abdullahu[3,*], Daniel O'Reilly[3,§], Philip J. Jensik[4], Masad J. Damha[3,†], and Keith T. Gagnon[1,2,†]

[1] Department of Chemistry and Biochemistry, Southern Illinois University, Carbondale, IL, USA.
[2] Department of Biochemistry and Molecular Biology, School of Medicine, Southern Illinois University, Carbondale, IL, USA.
[3] Department of Chemistry, McGill University, Montreal, Canada.
[4] Department of Physiology, School of Medicine, Southern Illinois University, Carbondale, IL, USA.
[§] Current Address: RNA Therapeutics Institute, University of Massachusetts Medical School, Worcester, Massachusetts, USA.
*These authors contributed equally.
†To whom correspondence should be addressed: masad.damha@mcgill.ca, ktgagnon@siu.edu

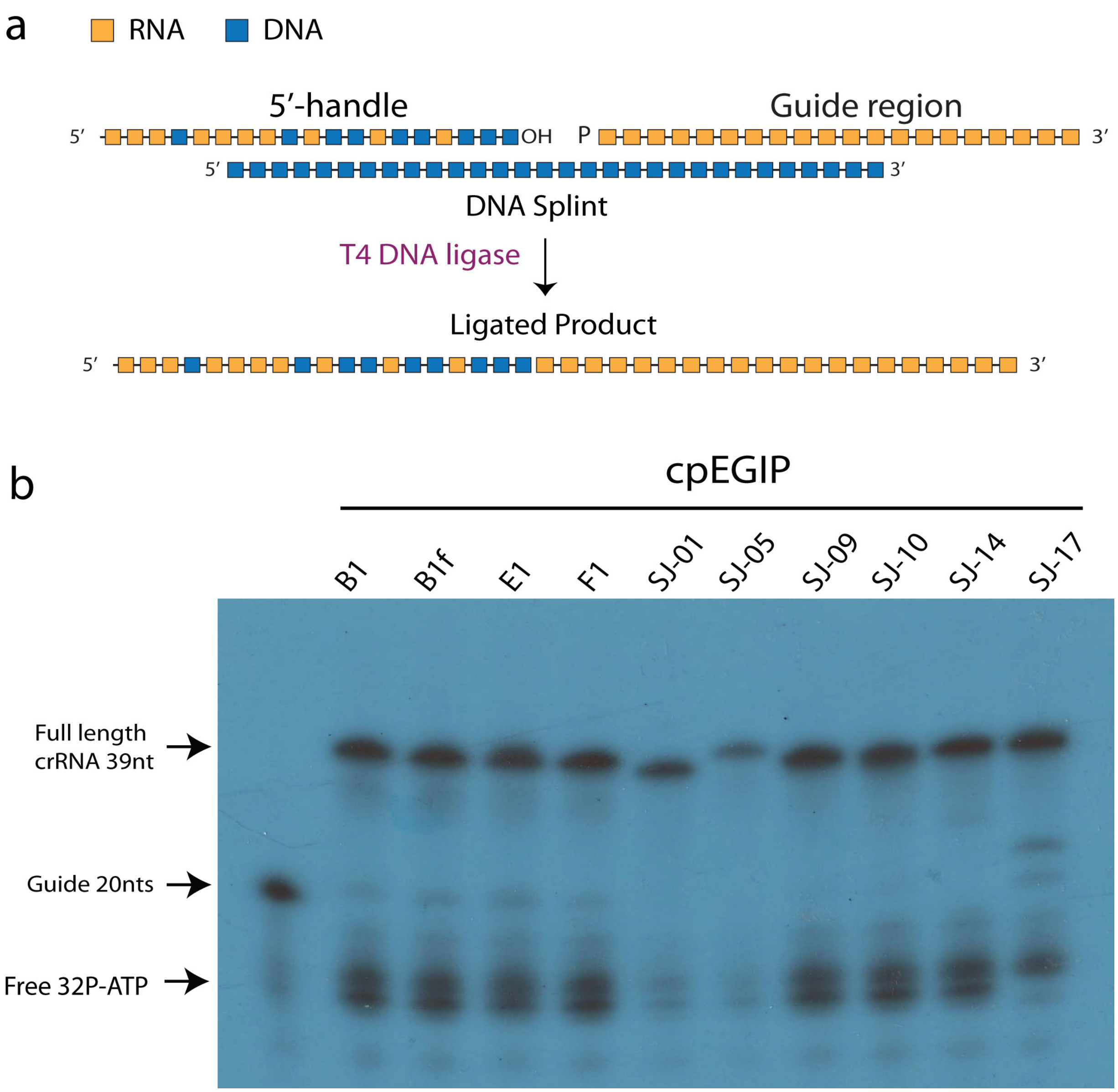

<u>Supplementary Figure 1</u>. Splint ligation of modified 5' handle pseudoknots and guide RNAs. (**A**) Illustration of ligation to make full-length crRNAs from chemically-modified 5' handles and 3' guides using a DNA splint and T4 DNA ligase. (**B**) Example gel where the guide RNA was 5'-radiolabeled to enable tracking of ligation product formation. Standard ligations did not use radioactivity but methylene blue staining instead. Full-length crRNA product was gel-purified for subsequent experiments as described in *Methods*.

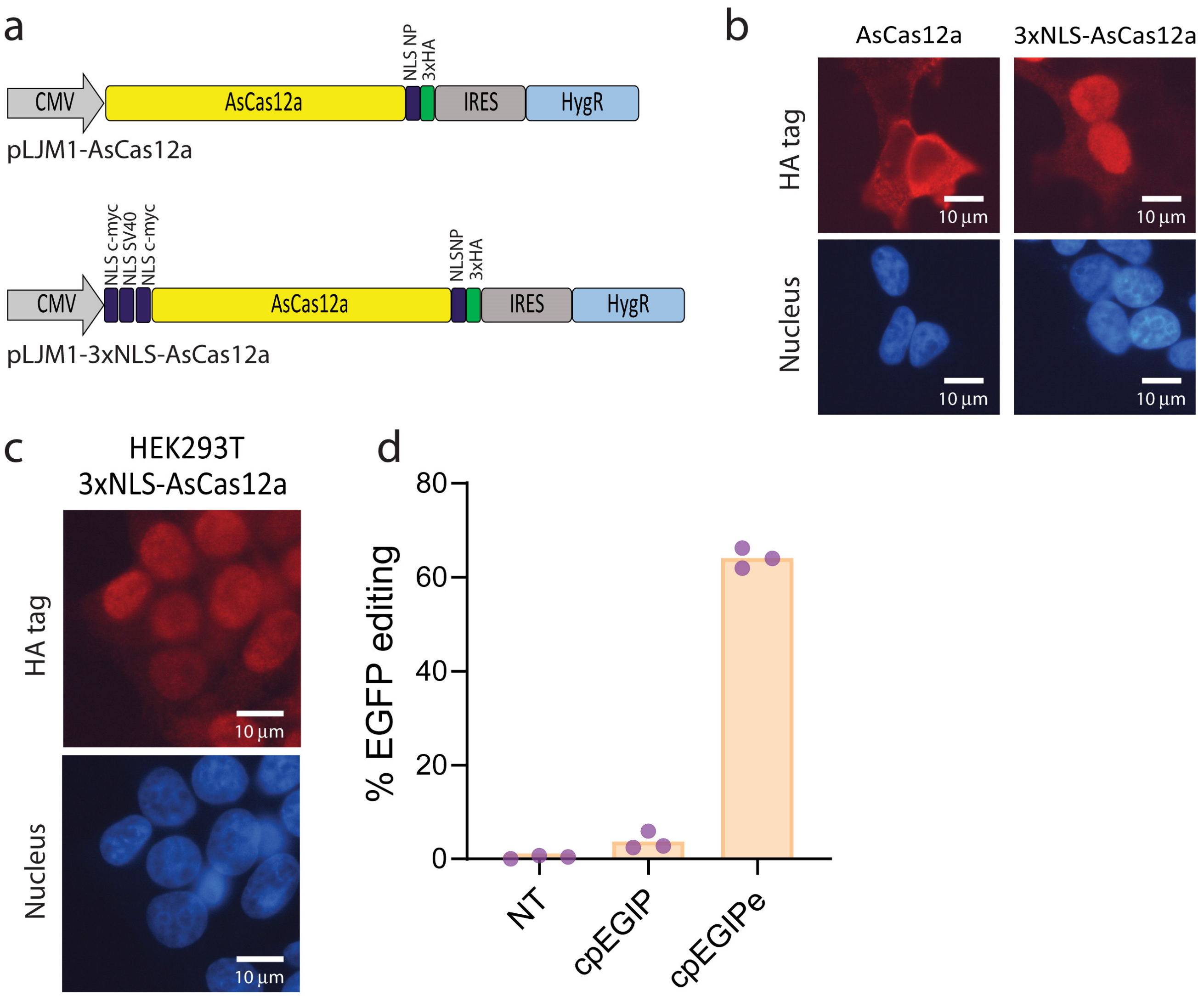

<u>Supplementary Figure 2</u>. Generation of *As*Cpf1 stable HEK+EGFP cells. (**A**) Two lentivectors expressing *As*Cpf1 were created, pLJM1-*As*Cpf1 (top) and pLJM1-3xNLS-*As*Cpf1 (bottom). Upon lentivirus infection, *As*Cpf1 expressed from pLJM1-*As*Cpf1 was observed to be primarily localized to the cytoplasm as determined by staining with an anti-HA antibody and immunofluorescence, indicating that the single NLS tag from the parent Addgene vector was not sufficient (**B**). Upon addition of a new 3x NLS tag at the N-terminus of *As*Cpf1, we observed robust nuclear localization (**B**) when expressed from this new vector, pLJM1-3xNLS-*As*Cpf1. Stable cell lines were then selected using hygromycin and these retained strong nuclear localization (**C**). Testing of two crRNA guides identified one (cpEGIPe) that generated high editing efficiency (**D**) when measured by loss of EGFP fluorescence in flow cytometry. The mean of three separate replicates (n = 3) is shown. Source data are provided as a Source Data file.

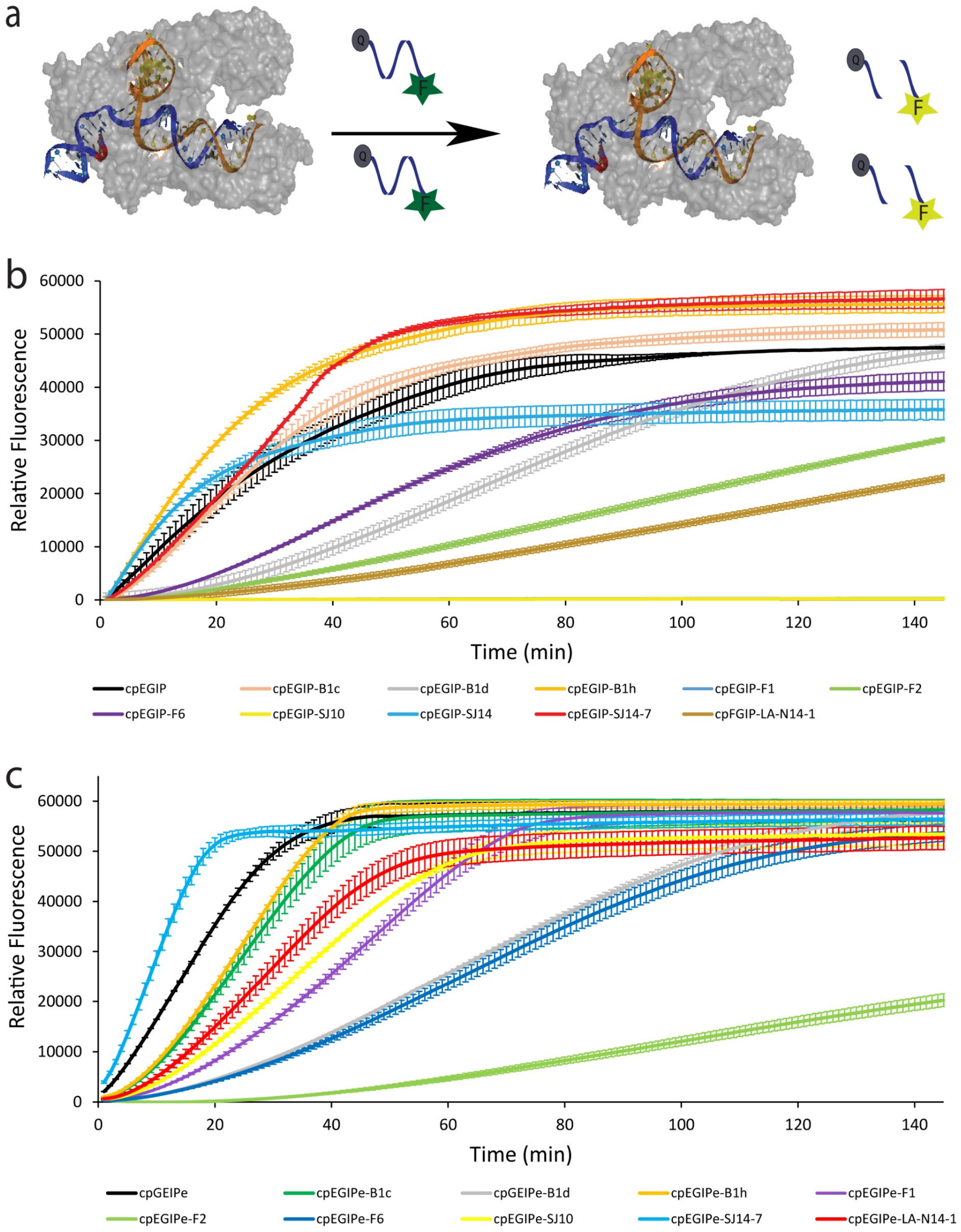

<u>Supplementary Figure 3</u>. **Fluorescence-based *trans* cleavage assay to measure non-sequence-specific ssDNase activity of *As*Cpf1.** (**A**) Illustration of *trans* cleavage assay, where a ssDNA bearing a fluorophore on one end and a quencher on the other is cleaved to unquench the fluorescence emission from the attached fluorophore (see *Methods*). (**B**) Representative time course data from the *trans* cleavage assay using the cpEGIP guide or (**C**) the cpEGIPe guide. Curves represent the mean of 3 separate replicates (n = 3). Error bars are S.E.M. Source data are provided as a Source Data file.

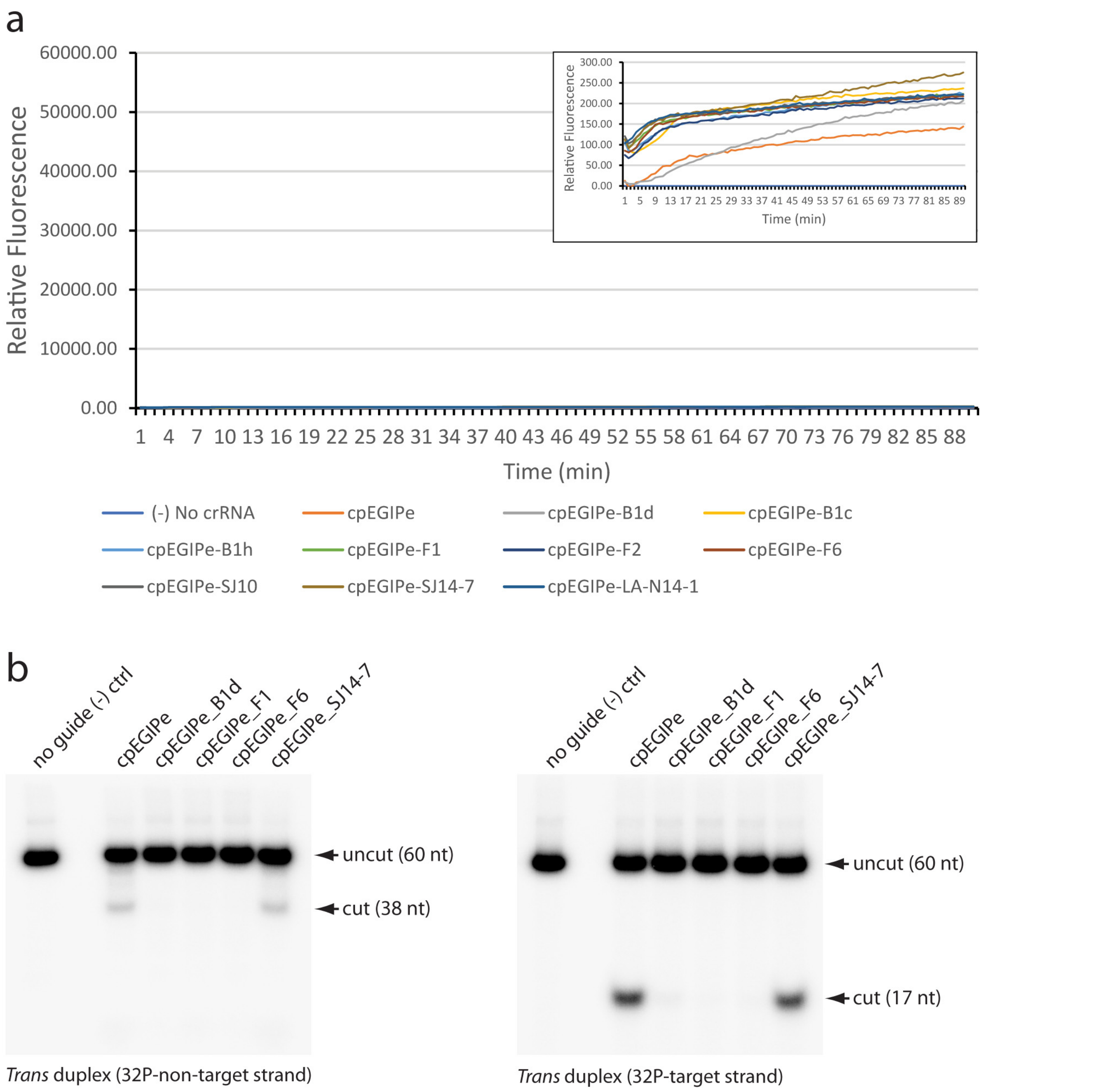

Supplementary Figure 4. Chemically modified pseudoknot-containing crRNAs do not elicit *trans* cleavage without target dsDNA and do not induce dsDNA nicking. (**A**) *Trans* cleavage assays using the indicated chemically modified cpEGIPe crRNAs but lacking a DNA target. No significant trans cleavage activity was observed in the absence of a DNA target. Inset shows fluorescence values at very small y-axis values. Three separate replicates (n = 3) were performed. (**B**) Duplex target DNA (60 bp) with a 5'-radiolabeled non-target (sense) strand (left) or 5'-radiolabeled target (antisense) strand (right) was included in typical *trans* cleavage assay conditions (see *Methods*) and cleavage products resolved by denaturing urea-PAGE after 60 min. Modified crRNAs are indicated above each lane and substrate and product DNA bands are indicated to the right of each gel. To ensure radiolabeled strands were completely double-stranded, each was annealed with an ~25% molar excess of unlabeled complement. Cleavage of radiolabeled strands mirrored previously quantified *cis* cleavage activity and did not indicate any substantial strand nicking by the selected crRNAs. Source data are provided as a Source Data file.

a

>EGIPe non-target (sense)_PS

5′ - GTAATTCTCCTTGGAATTTGCGTCGCCGTCCAGCT*C*G*A* | C*C*T*GGTTCATTCTCAAGCCTCA - 3′

>EGIPe target (antisense)_PS

5′ - TGAGGCTTGAGAAT*G*A*A* | C*C*A*GGTCGAGCTGGACGGCGACGCAAATTCCAAGGAGAATTAC - 3′

b

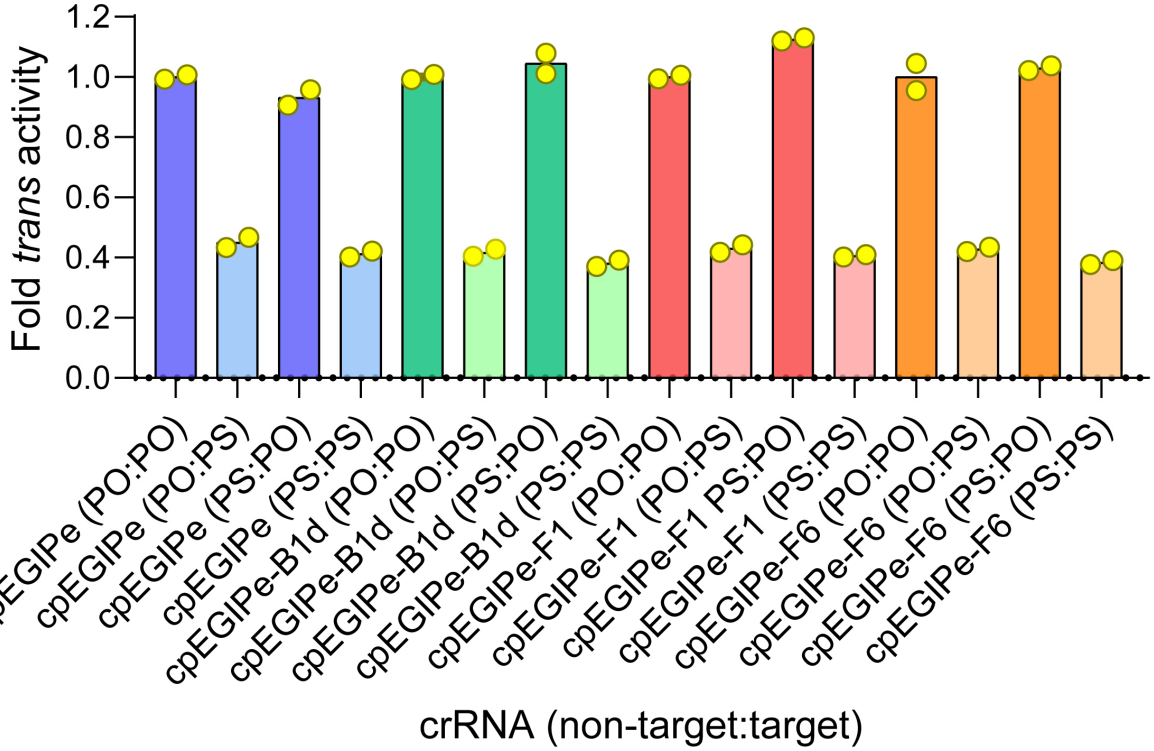

__Supplementary Figure 5__. **Phosphorothioate bonds at and surrounding the cleavage site of target DNA in the antisense strand impairs *trans* activity to the same degree for native and modified pseudoknots.** (**A**) Sequence and modification scheme for phosphorothioate-modified DNA target strands. Phosphorothioate (PS) bonds are indicated as blue asterisks. The expected position of cleavage is indicated with a red line. (**B**) Relative fold *trans* activity for native unmodified crRNA and crRNAs with modified pseudoknots taken after 120 min of reaction time. Activity is normalized to unmodified target DNA (PO:PO) for each crRNA. The modification for the sense strand is indicated first, then the antisense strand second. For example, PO:PS indicates a native phosphodiester (PO) sense strand and phosphorothioate (PS) antisense strand. The mean of two separate replicates (n = 2) is shown. Source data are provided as a Source Data file.

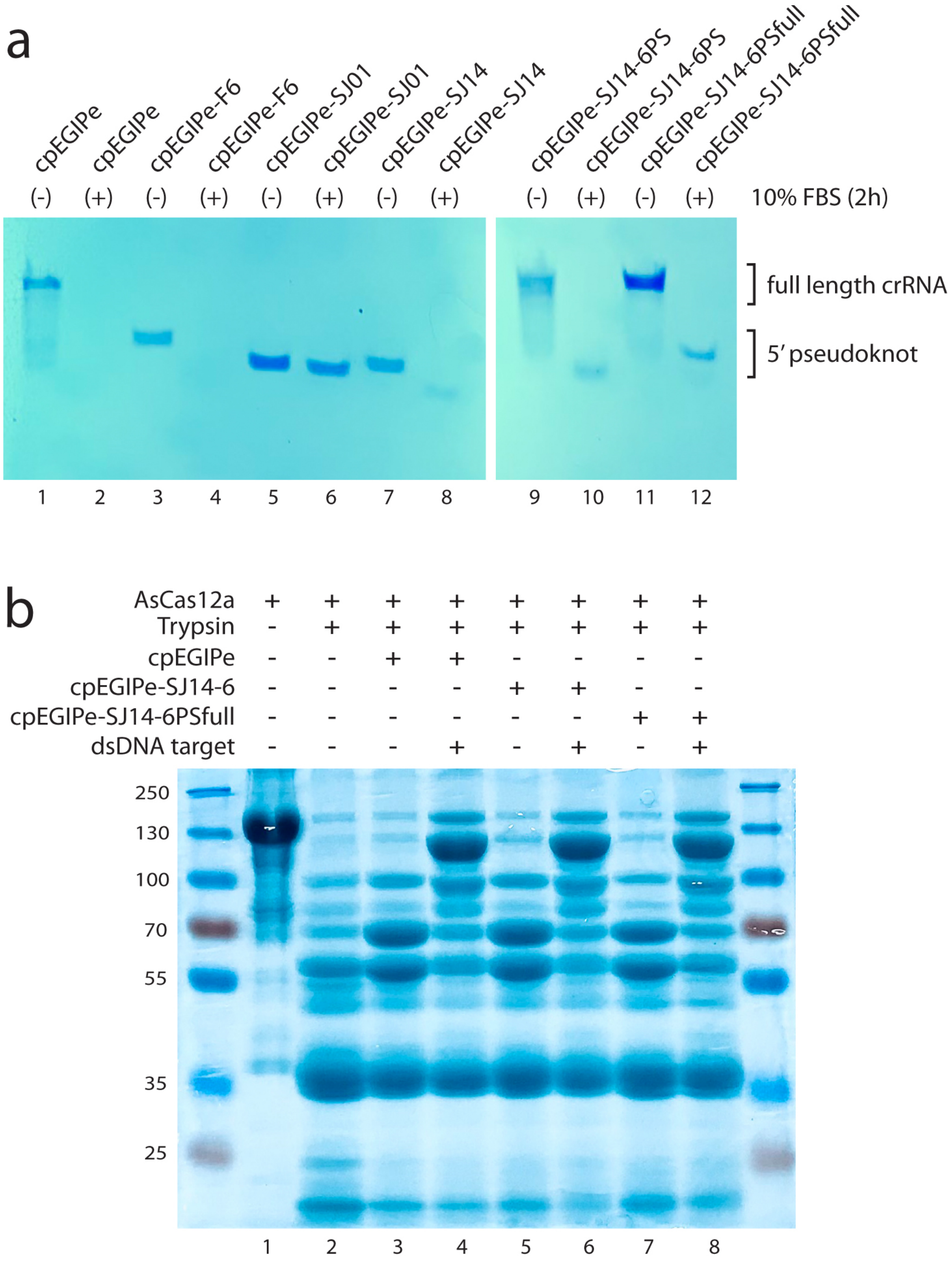

<u>**Supplementary Figure 6**</u>**. Effects of chemically-modified 5' pseudoknots on Cas12a structure and *in vitro* serum stability.** (**A**) *In vitro* serum stability of modified crRNA 5' pseudoknots only (lanes 1-8) or full-length crRNAs with modified 5' pseudoknots (lanes 9-12). RNA was incubated in 10% fetal bovine serum (FBS) for 2 h then resolved by denaturing polyacrylamide gel electrophoresis (PAGE) and stained with methylene blue. (**B**) Probing structural perturbations in *As*Cas12a induced by crRNA cpEGIPe, cpEGIPe-SJ14-6 and cpEGIPe-SJ14-6PSfull by limited trypsin hydrolysis. Proteolytic fragments were resolved by SDS-PAGE and stained with Coomassie blue.

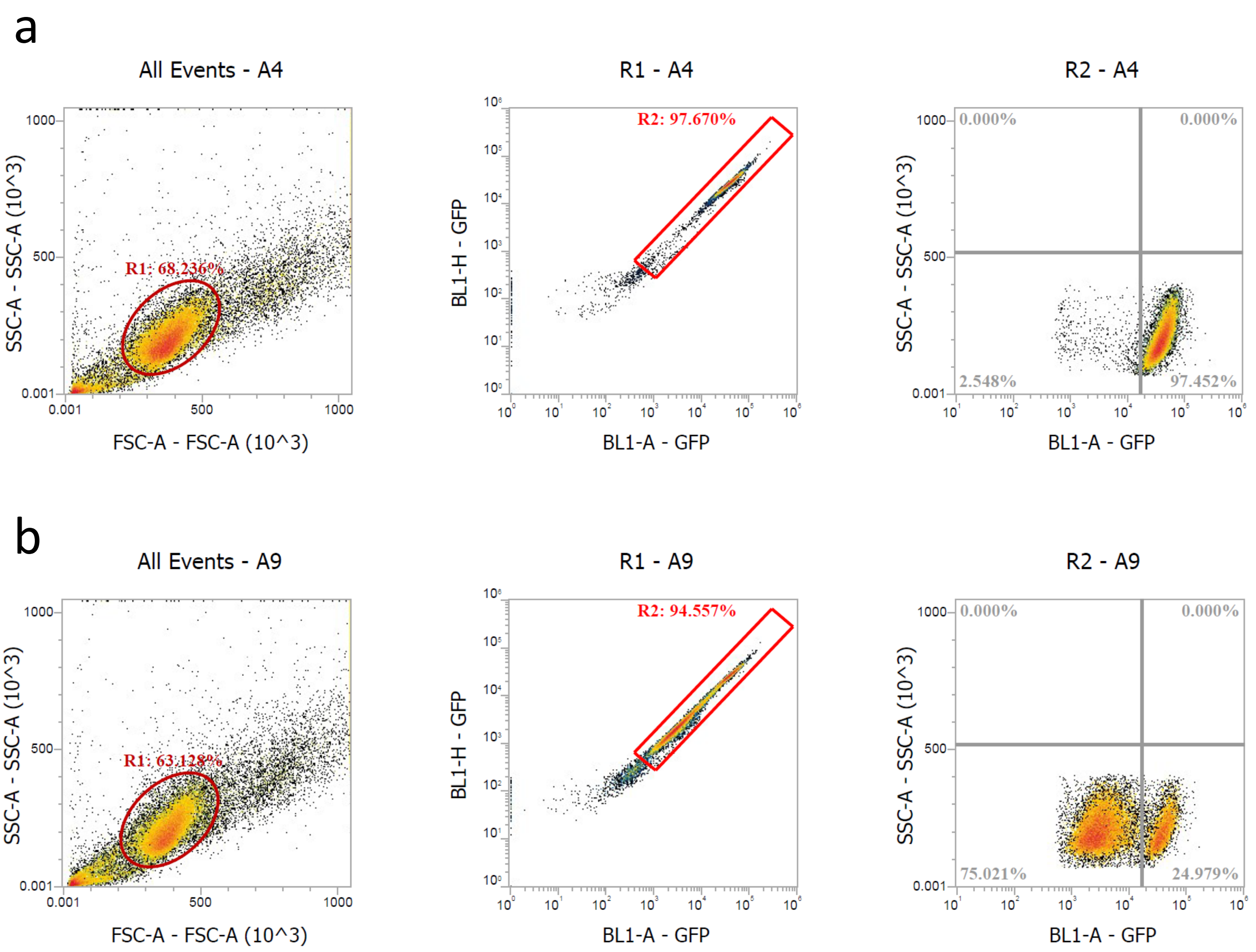

<u>Supplementary Figure 7</u>. Flow cytometry gating strategy for quantification of EGFP editing. (**A**) Gating for untreated (EGFP positive) cells and (**B**) for cells treated with CRISPR-Cas12a targeting EGFP (knockout) 5 days post transfection. Side scatter versus forward scatter are shown, along with percentages of cells that are gated as EGFP positive or negative.

Supplementary Table 1. Oligonucleotide Sequences.

cpEGIP_PO4-guide	/5Phos/rCrCrCrUrUrUrUrUrGrArGrUrUrUrGrGrArUrCrU
cpEGIPe_PO4-guide	/5Phos/rCrGrUrCrGrCrCrGrUrCrCrArGrCrUrCrGrArCrC
5' handle PK	rArArUrUrUrCrUrArCrUrGrUrUrGrUrArGrArU
cpEGIP guide-handle splint	CAAACTCAAAAAGGGATCTACAAGAGTAGA
cpEGIPe guide-handle splint	AGCTGGACGGCGACGATCTACAAGAGTAGA
cpEGIP_vitro targ_s	GTAATTCTCCTTGGAATTTGCCCTTTTTGAGTTTGGATCTTGGTTCATTCTCAAGCCTCA
cpEGIP_vitro targ_as	TGAGGCTTGAGAATGAACCAAGATCCAAACTCAAAAAGGGCAAATTCCAAGGAGAATTAC
cpEGIPe_vitro targ_s	GTAATTCTCCTTGGAATTTGCGTCGCCGTCCAGCTCGACCTGGTTCATTCTCAAGCCTCA
cpEGIPe_vitro targ_as	TGAGGCTTGAGAATGAACCAGGTCGAGCTGGACGGCGACGCAAATTCCAAGGAGAATTAC
ssDNA-FQ5 reporter	5IABKFQ/TTATT/36-FAM
cpEGIP_vitro targ_F	ATGCGATGGAGTTTCCCCA
cpEGIP_vitro targ_R	CCGCTTTACTTGTACAGCTCG

rN= RNA, 36-FAM= 3' 6-FAM, 5IABKFQ= 5' Iowa Black Quencher

Supplementary Table 2. HRMS data of synthesized oligonucleotides with predicted and observed masses.

Sample Name	Predicted mass	Observed mass
cpEGIP-SJ01	5980.6744	5980.7500
cpEGIP-SJ03	6058.8379	6058.9063
cpEGIP-SJ04	6058.8379	6058.9300
cpEGIP-SJ05	6058.8379	6058.9300
cpEGIP-SJ06	5942.7531	5942.7831
cpEGIP-SJ09	5974.6873	5974.7500
cpEGIP-SJ10	5976.6830	5976.7188
cpEGIP-SJ12	5974.6874	5974.6563
cpEGIP-SJ13	5976.6831	5976.6250
cpEGIP-SJ14	5966.7037	5966.6719
cpEGIP-SJ15	5972.6916	5972.6875
cpEGIP-SJ16	5972.6916	5970.7188
cpEGIP-SJ17	5970.7209	5969.7344
cpEGIP-SJ14-1	6008.7851	6008.8672
cpEGIP-SJ14-2	6008.8016	6008.8672
cpEGIP-SJ14-3	6008.7859	6008.8672
cpEGIP-SJ14-4	6008.8016	6008.8672
cpEGIP-SJ14-5	6008.8016	6008.8672
cpEGIP-SJ14-6	6008.7860	6008.8672
cpEGIP-SJ14-7	6008.8016	6008.8672
cpEGIP-LA-N14-1	6017.8295	6017.7734
cpEGIP-LA-N14-2	6017.8486	6014.6875
cpEGIP-LA-N14-3	6017.8295	6017.7734
cpEGIP-LA-N14-4	6017.8295	6017.7734
cpEGIP-LA-N14-5	6017.8295	6017.7734
cpEGIP-LA-N14-6	6005.8047	6005.6875
cpEGIP-LA-N14-7	6005.8081	6005.6875
cpEGIP-LA-N14-8	6005.8047	6005.6875
cpEGIP-LA-N14-9	6005.8047	6005.6875
cpEGIP-LA-N14-10	6005.8047	6005.6875
cpEGIPe	12300.2573	12371.4081
epEGIPe-SJ10	12334.8180	12333.5000
cpEGIPe-F1	12192.4253	12190.8355
cpEGIPe-F6	12176.4263	12175.1959
