# Supplementary material for: Gene editing with CRISPR-Cas12a guides possessing ribose-modified pseudoknot handles
Source: Nat Commun. 2021 Nov 15;12:6591. doi: 10.1038/s41467-021-26989-z (PMC8593028; doi:10.1038/s41467-021-26989-z)
Supplement: Supplementary file 2 — Reporting summary. [file 41467_2021_26989_MOESM2_ESM.pdf]

nature portfolio

Corresponding author(s): Keith T. Gagnon

Last updated by author(s): Oct 11, 2021

Reporting Summary

Nature Portfolio wishes to improve the reproducibility of the work that we publish. This form provides structure for consistency and transparency in reporting. For further information on Nature Portfolio policies, see our Editorial Policies and the Editorial Policy Checklist.

Statistics

For all statistical analyses, confirm that the following items are present in the figure legend, table legend, main text, or Methods section.

n/a	Confirmed	
☐	☒	The exact sample size (*n*) for each experimental group/condition, given as a discrete number and unit of measurement
☐	☒	A statement on whether measurements were taken from distinct samples or whether the same sample was measured repeatedly
☐	☒	The statistical test(s) used AND whether they are one- or two-sided<br>*Only common tests should be described solely by name; describe more complex techniques in the Methods section.*
☒	☐	A description of all covariates tested
☒	☐	A description of any assumptions or corrections, such as tests of normality and adjustment for multiple comparisons
☐	☒	A full description of the statistical parameters including central tendency (e.g. means) or other basic estimates (e.g. regression coefficient) AND variation (e.g. standard deviation) or associated estimates of uncertainty (e.g. confidence intervals)
☒	☐	For null hypothesis testing, the test statistic (e.g. *F*, *t*, *r*) with confidence intervals, effect sizes, degrees of freedom and *P* value noted<br>*Give P values as exact values whenever suitable.*
☒	☐	For Bayesian analysis, information on the choice of priors and Markov chain Monte Carlo settings
☒	☐	For hierarchical and complex designs, identification of the appropriate level for tests and full reporting of outcomes
☒	☐	Estimates of effect sizes (e.g. Cohen's *d*, Pearson's *r*), indicating how they were calculated

Our web collection on statistics for biologists contains articles on many of the points above.

Software and code

Policy information about availability of computer code

Data collection	No custom software or code were used in this manuscript. Commercial software was used for data analysis, which included Attune NxT Analysis (v3.12), ImageJ (v1.43u), IDT OligoAnalyzer tool (https://www.idtdna.com/calc/analyzer), and GraphPad Prism (v9).
Data analysis	No custom software were used for data collection.

For manuscripts utilizing custom algorithms or software that are central to the research but not yet described in published literature, software must be made available to editors and reviewers. We strongly encourage code deposition in a community repository (e.g. GitHub). See the Nature Portfolio guidelines for submitting code & software for further information.

Data

Policy information about availability of data

All manuscripts must include a data availability statement. This statement should provide the following information, where applicable:

- Accession codes, unique identifiers, or web links for publicly available datasets
- A description of any restrictions on data availability
- For clinical datasets or third party data, please ensure that the statement adheres to our policy

No large bioinformatic, sequencing, or quantitative data sets are associated with this study. All relevant quantifiable data for enzyme activity are presented. Original and processed data sets can be made available upon request and do not appear to fall under mandated sharing.

Field-specific reporting

Please select the one below that is the best fit for your research. If you are not sure, read the appropriate sections before making your selection.

- [x] Life sciences
- [ ] Behavioural & social sciences
- [ ] Ecological, evolutionary & environmental sciences

For a reference copy of the document with all sections, see nature.com/documents/nr-reporting-summary-flat.pdf

Life sciences study design

All studies must disclose on these points even when the disclosure is negative.

Sample size	No sample size calculations were performed. It is our experience that two biological replicates for in vitro cleavage assays by CRISPR-Cas12a are highly reproducible and for cell-based editing 4-6 replicates are appropriate based on previous experience by our lab and others.
Data exclusions	no data were excluded from this study.
Replication	All samples were repeated as biological replicates the number of times indicated in figure legends or methods section.
Randomization	Randomization of samples was not performed. There were no preconceived notions as to which modified guide designs would work or not and the order of testing would not have conceivably altered results with our assays.
Blinding	Blinding was not necessary since there were no preconceived notions as to which guide designs would work. The enzyme activity data from these studies is very objective and cannot be easily manipulated by the researcher's perception of the study outcomes.

Reporting for specific materials, systems and methods

We require information from authors about some types of materials, experimental systems and methods used in many studies. Here, indicate whether each material, system or method listed is relevant to your study. If you are not sure if a list item applies to your research, read the appropriate section before selecting a response.

Materials & experimental systems

n/a	Involved in the study	
	x	Antibodies
	x	Eukaryotic cell lines
x		Palaeontology and archaeology
x		Animals and other organisms
x		Human research participants
x		Clinical data
x		Dual use research of concern

Methods

n/a	Involved in the study	
x		ChIP-seq
	x	Flow cytometry
x		MRI-based neuroimaging

Antibodies

Antibodies used	anti-HA (Santa Cruz Biotechnology) (sc-7392)
Validation	Validation in cell-based systems very similar to ours (HEK293T cells) is strongly suggested on the manufacturer's website (https://www.scbt.com/p/ha-probe-antibody-f-7?gclid=Cj0KCQjwwY-LBhD6ARIsACvT72MsHF9xpD3O8GQsUJG5ZNbZC5E_YncVpavGGiZMs_k789vtZst3jrMaAjRPEALw_wcB) based on 4.6 out of 5 stars from 119 user reviews. User feedback is the primary method of antibody validation for this reagent.

Eukaryotic cell lines

Policy information about cell lines

Cell line source(s)	HEK 293T + EGFP, kind gift from Dr. Wen Xue, UMass Medical School
Authentication	See manuscript reference Yin et al., 2017. Briefly, cells were quantified for fluorescence using flow cytometry and found to possess >95% positive cells. Cells were genotyped prior to making stable with lentivirus.
Mycoplasma contamination	Negative.
Commonly misidentified lines (See ICLAC register)	No commonly misidentified cell lines were used in this study.

Flow Cytometry

Plots

Confirm that:

☒ The axis labels state the marker and fluorochrome used (e.g. CD4-FITC).

☒ The axis scales are clearly visible. Include numbers along axes only for bottom left plot of group (a 'group' is an analysis of identical markers).

☒ All plots are contour plots with outliers or pseudocolor plots.

☒ A numerical value for number of cells or percentage (with statistics) is provided.

Methodology

Sample preparation	Cells were reverse transfected (40,000 cells) in six experimental replicates in 96-well plates with 20 pmols of crRNA and 0.3 µL RNAiMAX lipid (Invitrogen) in a final reaction of 200 µL of OptiMEM. After 8 h, one volume of media containing 5% FBS and 5% CCS was added to cells and further incubated overnight. Media was then replaced with full media and cells grown for an additional 4 days. For flow cytometry, cells were washed with 200 µL PBS and trypsinized by adding 70 µL of trypsin-EDTA solution. 100 µL of media was added to the cell. The cells were spun for 5 min at 300 x g at room temperature. Cells were washed again with 200 µL PBS, resuspended in 200 µL PBS and counted in an Attune flow cytometer.
Instrument	Attune NxT
Software	Attune NxT Software v3.1.2
Cell population abundance	initial cell population was >95 EGFP expressing. After sorting, the EGFP positive cells depended upon degree of CRISPR knockout. At least 20,000 events (cells) were collected (counted) and analyzed.
Gating strategy	EGFP was detected using the blue laser (BL1 channel). At least 20,000 events were collected and analyzed by Attune software (v3.12). The cells were gated based on forward and side scattering (FSC-A/SSC-A) to remove cell debris, gated to select single cells, and gated to select EGFP positive cells. The quadrant gate was established using the signal from non-EGFP expressing control cells. Untreated HEK293T cells expressing EGFP and AsCas12a contained ~5% non-fluorescent cells (Figure S7).

☒ Tick this box to confirm that a figure exemplifying the gating strategy is provided in the Supplementary Information.
